# MMP3 at the crossroads: Linking molecular pathways to disease diagnosis and therapy

**DOI:** 10.1016/j.phrs.2025.107750

**Published:** 2025-04-30

**Authors:** Jing Jiang, Qiong Wu, Snekha Rajasekaran, Rongxue Wu

**Affiliations:** a Section of Cardiology, Department of Medicine, Biological Sciences Division, University of Chicago, Chicago, IL, United States; b Binzhou Medical University, Yantai, China

**Keywords:** Matrix metalloproteinase 3 (MMP-3), Extracellular matrix (ECM), Inflammatory diseases, Infectious diseases, Cardiovascular diseases, Neurodegenerative diseases, Cancer

## Abstract

Matrix metalloproteinase 3 (MMP-3) is a multifaceted enzyme that plays a critical role in the regulation of extracellular matrix (ECM) dynamics, influencing both normal physiological and pathological processes. In addition to its established role in ECM degradation, MMP-3 is gaining recognition for modulating cellular behaviors such as inflammation, migration, and proliferation. Recent research has uncovered its capacity to activate latent signaling molecules, release growth factors from the ECM and interact with various cell surface receptors, linking MMP-3 to the progression of various diseases, including inflammatory diseases, infection diseases, cardiovascular diseases, neurodegenerative disorders, and cancer. The review provides an overview of MMP-3’s molecular regulation, emphasizing the mechanisms controlling its expression and activity. We discuss MMP3’s involvement in both ECM-dependent and independent pathways, and its potential as a diagnostic, prognostic biomarker in various diseases. Additionally, we explore therapeutic strategies targeting MMP-3, summarizing ongoing efforts to develop specific inhibitors and modulate its activity in different pathologic conditions. Through this review, we aim to consolidate the diverse functions of MMP-3 and provide new insights into future research directions, particularly in translating these findings into clinical applications.

## 1. Introduction

Matrix metalloproteinase 3 (MMP-3), also known as Stromelysin-1, localized to chromosome 11q22.3, is a member of the metalloproteinase enzyme family, which consists of approximately 20 zinc-dependent endopeptidases [1]. The matrix metalloproteinases (MMPs) family share common structural and functional elements and are products of different genes. There are at least 25 members in the MMP family, which are further divided into six subfamilies – the collagenases, gelatinases, matrilysins, stromelysins, metalloelastase and membrane-type MMPs based on their structure and substrate specificity [2]. The structure of MMP-3, like most members of the MMP family is organized into three basic, distinctive, and well conserved domains: an amino-terminal propeptide; a catalytic domain; and a hemopexin-like domain at the carboxy-terminal [3]. The main role of MMP3 is involved in the breakdown of the extracellular matrix (ECM) proteins during tissue remodeling in normal physiological processes, such as embryonic development and reproduction [4]. The early studies on MMP-3 focused on its roles in collagen degradation and various pathological conditions, including arthritis, fibrosis, and cancer. For example, in arthritis, MMP-3 can be induced by inflammatory cytokines such as interleukin-1 (IL-1) and tumor necrosis factor-alpha (TNF-alpha) in rheumatoid synovium, degrades a number of extracellular matrix components of cartilage, and plays central roles in rheumatoid joint destruction [5,6]. MMP-3 also has been extensively studied in various kinds of cancer as well as cancer-to be-associated fibrosis, a common feature of many types of cancer, including breast, lung, and colon cancer [7–9]. Later, MMP-3 is found to be secreted by various cell types, including macrophages, fibroblasts, adipocytes, chondrocytes, endocrine cells, germ cells, muscle cells other than cancer cells and endothelial cells. And it not only plays a key role in the degradation of ECM components, including collagen, elastin, proteoglycans and other pro-MMPs [4,10], but also involved in inflammation as a pro-inflammatory factor, modulating the synthesis and the release of cytokines and chemokines, and in cell growth, proliferation, and remodeling [11,12]. MMP-3 has been extensively studied in various disease and disease models like the regulation of cellular responses to injury, including inflammation and tissue repair other than cancer, neurodegeneration, fibrosis, arthritis, obesity, cardiovascular diseases. And it has been identified as a potential therapeutic target for various diseases. This knowledge prompted the development of more than 50 MMP inhibitors investigated in clinical trials for cancer or arthritis indications as the first generation of broad- spectrum synthetic inhibitors. Unfortunately, all of these trials failed despite the wealth and promising preclinical data supporting the strong potential of the inhibitors could be one of therapeutics [13]. One of the main reasons for the failure has been stated as the poor knowledge of complexity of MMP functions together with the broad-spectrum inhibitors resulted in opposite effect or undesired side effects. The development of techniques such as NMR has greatly facilitated the design of specific MMP inhibitors, leading to the creation of an increasing number of second-generation, highly specific MMP inhibitors [14,15]. However, a thorough and systematic understanding of the fundamental biological functions of MMP-3 remains crucial as it serves as an important foundation for leveraging MMP-3 as an effective potential target for disease diagnosis and therapy. Primarily, the regulation of MMP-3 expression is key. Like other MMPs, MMP-3 are naturally regulated at any of the following three known levels: the transcription level (mRNA) including post-transcriptional level, the pro-enzyme activation level, and inhibition of the active forms by various tissue inhibitors of MMPs (TIMPs) [16]. The role of MMP-3 as an active molecule involves its participation in biological processes, including the regulation of other molecules, factors, and signaling pathways. A systematic understanding of these two aspects will clarify why MMP-3 is widely used as a biomarker or target for the treatment and/or diagnosis of various diseases. This review aims to categorize and summarize the applications and reports of MMP-3 as a target for disease treatment and/or diagnosis over the past few decades. While it may not exhaustively cover every specific disease, it broadly encompasses the major disease categories affected. In particular, it focuses on the shared molecular mechanisms through which MMP-3 contributes to fibrosis across key organ systems, rather than detailing each fibrotic condition. It also examines the regulation of MMP-3 expression and its direct and indirect involvement in disease-related biological processes, underscoring its therapeutic relevance.

## 2. Molecular regulation and function of MMP3

**Fig. 1.**
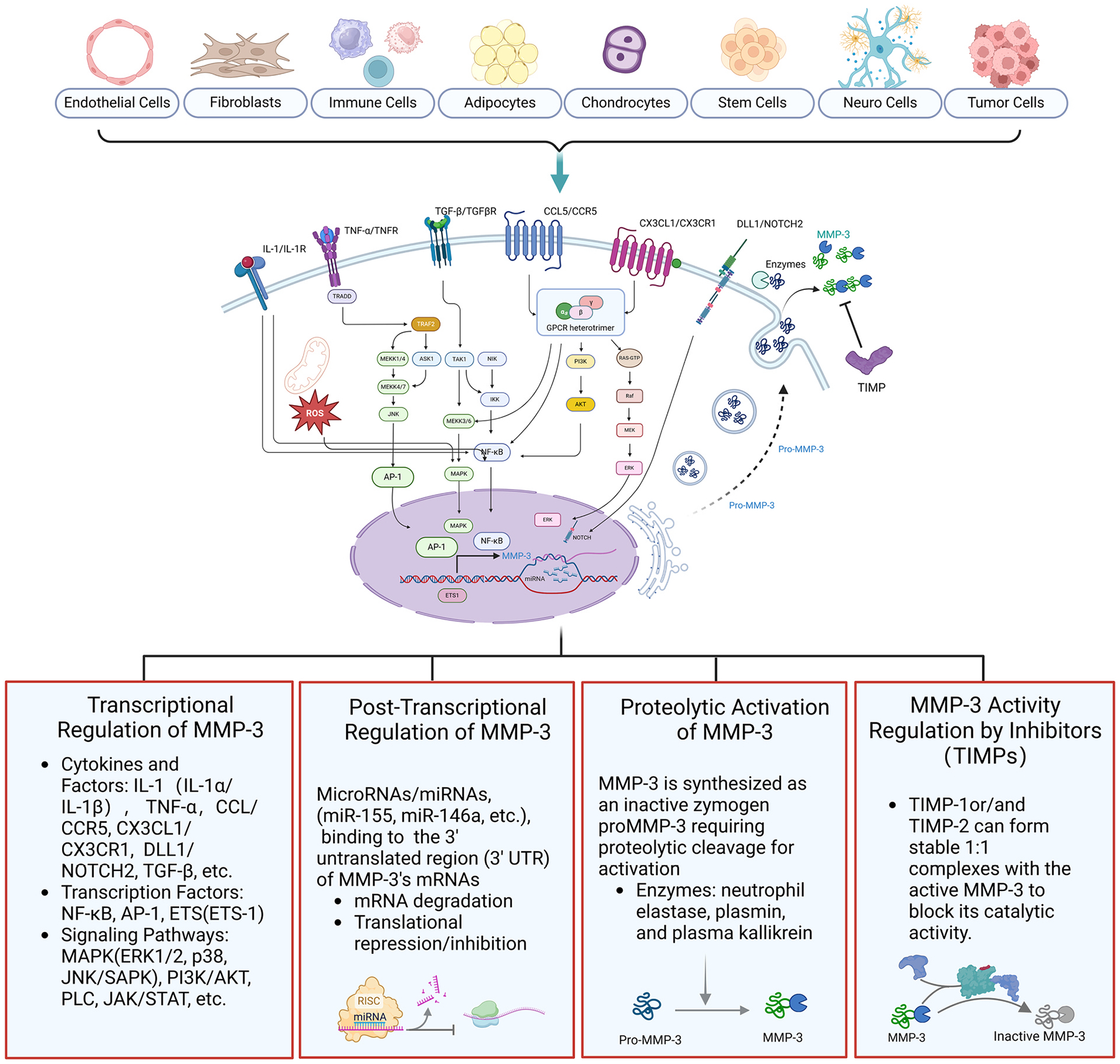
Schematic representation of the multifactorial regulation of MMP-3 in various cell types. This figure highlights four key aspects of MMP-3 regulation: transcriptional control, post-transcriptional modulation, proteolytic activation (zymogen activation), and endogenous inhibition (via TIMPs).*Abbreviations*: IL-1 = interleukin-1; IL-1R = interleukin-1 receptor; TNF-α = tumor necrosis factor alpha; TNFR = tumor necrosis factor receptor; TGF-β = transforming growth factor beta; TGFβR = transforming growth factor beta receptor; CCL = chemokine (C-C motif) ligand; CCR5 = C-C motif chemokine receptor 5; CX3CL = chemokine (C-X3-C motif) ligand; CX3CR = C-X3-C motif chemokine receptor; DLL = delta-like protein; NOTCH = neurogenic locus notch homolog protein; TRADD = TNF receptor type 1-associated death domain protein; TRAF2 = TNF receptor-associated factor 2; GPCR = G-protein coupled receptor; MEKK = mitogen-activated protein kinase kinase kinase (MAP3K); MEK = mitogen-activated protein kinase kinase; ASK1 = apoptosis signal-regulating kinase 1 (MAP3K5); TAK1 = mitogen-activated protein kinase kinase kinase 7 (MAP3K7); MAPK = mitogen-activated protein kinase; JNK = c-Jun N-terminal kinase; NF-κB = nuclear factor kappa-light-chain-enhancer of activated B cells; NIK = NF-kappa-B-inducing kinase; IKK = Ikappa B kinase; PI3K = phosphoinositide 3-kinase; AKT = Protein kinase B (PKB); RAS = Rat sarcoma virus, small GTPase; RAF = rapidly accelerated fibrosarcoma, serine/threonine-specific protein kinase; ERK = extracellular signal -regulated kinase; AP-1 = activator protein 1; ETS1 = protein C-ets-1, ETS family of transcription factor; MMP-3 = matrix metalloproteinase 3; miRNA = micro-RNA.

MMP-3, expressed in various cell types, plays a central role in degrading ECM components, which is essential for normal physiological processes such as embryogenesis, tissue repair, and wound healing. However, dysregulation of MMP-3 has been linked to pathological conditions, including inflammation, fibrosis, cardiovascular diseases, neurodegeneration, and cancer etc. The regulation of MMP-3 occurs at multiple levels, encompassing transcriptional, post-transcriptional, and proteolytic activation, as well as inhibition by endogenous tissue inhibitors of metalloproteinases (TIMPs) (Fig. 1). These tightly controlled regulatory mechanisms ensure that MMP-3 activity is modulated to meet the needs of specific cellular environments, preventing unwarranted tissue damage.

### 2.1. Transcriptional regulation

Transcriptional regulation of MMP-3 is influenced by various signaling molecules, primarily pro-inflammatory cytokines, growth factors, and hormones, which activate different intracellular signaling pathways [17–21]. These pathways, in turn, interact with the cis-regulatory elements present in the MMP-3 promoter region, driving its transcription in a tissue- and context-specific manner. The most well-studied inducers of MMP-3 are cytokines such as IL-1, tumor necrosis factor-alpha (TNF-α), and transforming growth factor-beta (TGF-β), all of which play key roles in the immune response and tissue remodeling.

#### 2.1.1. IL-1, TNF-α and other factors-mediated pathways

IL-1, particularly IL-1α and IL-1β, upregulates MMP-3 transcription through several signaling pathways, including the mitogen-activated protein kinase (MAPK) pathway, nuclear factor kappa-light-chain-enhancer of activated B cells (NF-κB), and the activator protein-1 (AP-1) transcription factor [22]. IL-1α stimulates protein kinase C (PKC) and p38 MAPK, leading to the activation of AP-1, which binds to specific sites in the MMP-3 promoter region to initiate transcription [23]. Additionally, IL-1β stimulates MMP-3 transcription by generating reactive oxygen species (ROS), which in turn activates the MAPK and NF-κB pathways. NF-κB translocates to the nucleus, where it binds to the MMP-3 promoter and induces its transcription [24–26].

TNF-α, another potent pro-inflammatory cytokine, operates through similar mechanisms, activating both NF-κB and AP-1 to promote MMP-3 transcription. TNF-α can also engage the Notch signaling pathway [27]. For example, the interaction of Notch ligand delta-like protein 1 (DLL-1) with Notch-2 in joint macrophages enhances the production of MMP-3 and pro-inflammatory cytokines, including IL-6 [28,29]. Additionally, MMP-3 and other inflammatory cytokines upregulated by TNF-α promoted the upregulation of interferon-stimulated genes is mechanistically due to ERK1/2 and Janus Kinase(JAK)-STAT1 pathways in the model of age-to beassociated B cells (ABCs) involved rheumatoid arthritis autoimmune disease, where elevated levels of MMP-3 contribute to tissue destruction by degrading collagen and other ECM components [30,31].

Other factors engaged in regulation role in MMP-3 expression like Bmi-1, a member of Polycom Group, was also reported in malignant gliomas [32]. In human chondrosarcoma tissues, CCL5 upregulate MMP-3 expression via activation of phosphatidylinositol 3-kinase (PI3K), Akt and NF-kappaB pathways after binding its receptor CCR5 [33]. In an osteoarthritis pathogenesis model, under the CX3CL1 stimulation and binding to its receptor CX3CR1, MMP-3 transcription is synergistically regulated by NF-kappaB with other cytoplastic downstream signal pathway factors (e.g. C-Raf, MEK, ERK) [34].

#### 2.1.2. TGF-β and its dual role

TGF-β exhibits a more complex, context-dependent regulatory role in MMP-3 expression. In some cell types, TGF-β induces MMP-3 transcription through the Smad signaling pathway, promoting ECM remodeling and fibrosis. A computational study combined with virtual laboratory investigation found that the complex of Smad4-DNA fragment has high binding affinity and stability with common single nucleotide polymorphisms (SNPs) of MMP3 promoter motif, which alters the gene expression of MMP-3 [35]. This pathway is often activated in response to tissue injury, where TGF-β plays a key role in wound healing by facilitating the degradation and replacement of damaged ECM [36]. However, under different conditions, TGF-β can repress MMP-3 expression, particularly when it functions in concert with other cytokines, demonstrating the multifaceted nature of TGF-β’s interaction with MMP-3 [37,38]. In physiological processes like tissue repair, TGF-β upregulates MMP-3 to facilitate ECM remodeling and tissue regeneration. However, prolonged MMP-3 activity may lead to excessive ECM degradation and tissue destruction. Conversely, TGF-β-mediated repression of MMP-3 maintains ECM integrity and prevents uncontrolled matrix breakdown in tissue homeostasis. In pathological conditions such as fibrosis and cancer, TGF-β-induced upregulation of MMP-3 can promote excessive ECM turnover, contributing to fibrosis progression and tumor metastasis. In contrast, insufficient TGF-β repression of MMP-3 may exacerbate ECM degradation and tissue damage. Therefore, the dual regulation of MMP-3 by TGF-β plays a crucial role in balancing tissue remodeling and pathology.

#### 2.1.3. Transcription factors involved

Apart from cytokine-mediated regulation, several transcription factors directly regulate MMP-3 expression. Besides TGF-β which has been discussed above, key transcription factors involved in MMP-3 expression include NF-κB, AP-1and ETS family etc. These factors integrate signals from inflammation, tissue injury, and cellular stress to modulate MMP-3 expression in a context-dependent manner.

NF-κB, as mentioned, is a key mediator of the inflammatory response and a major regulator of MMP-3 transcription. After activated by various pro-inflammatory cytokines as well as environmental factors, NF-κB leads to its translocation to the nucleus where it binds to specific response elements in the MMP-3 promoter region, thereby initiating transcription. The polymorphic element in the MMP-3 promoter provides additional mechanisms for gene regulation by influencing transcription factor binding. [39]. For example, the 5 T/6 T polymorphism in the MMP-3 promoter affects its regulation by NF-κB and zinc-binding protein 89 (ZBP-89), which compete for binding in a polymorphism-specific manner. This competition is believed to have physiological effects that depend on the relative abundance and activity of these factors in different cell types and conditions. [40]. ROS, key environmental factors produced both endogenously and exogenously, play a pivotal role in regulating MMP-3 expression within tumor microenvironmental cells through the activation of NF-κB pathway [41]. In inflammatory diseases, such as rheumatoid arthritis (RA), NF-κB-mediated upregulation of MMP-3 facilitates joint destruction through enhanced matrix degradation.

AP-1, a dimeric transcription factor composed of proteins from the Fos and Jun families, also plays a crucial role. It binds to the AP-1 binding site in the MMP-3 promoter, driving transcription in response to various extracellular signals [42,43]. Importantly, AP-1-mediated regulation is subject to fine-tuning by other cytokines, such as IL-4, which can inhibit MMP-3 expression under certain pathological conditions such as fibrosis and cancer. Overactivation of AP-1 may drive excessive MMP-3 expression, leading to pathological ECM remodeling and tissue damage. Other transcription factors, such as members of the erythroblast transformation-specific (ETS) family, also contribute to MMP-3 regulation [44,45].

ETS1, for example, is known to bind to ETS binding sites located near the AP-1 sites in the MMP-3 promoter, further enhancing transcription [46,47] through palindromic head-to-head ETS binding sites (EBS) [48]. This collaborative interaction between different transcription factors allows for precise control of MMP-3 expression in response to diverse cellular stimuli.

The mechanisms primarily responsible for regulating MMP-3 transcription under different physiological and/or pathophysiological conditions include the activation of these transcription factors by inflammatory cytokines, stress signals, and growth factors. Under physiological conditions, the expression of MMP-3 is finely tuned to balance ECM turnover and tissue integrity. In wound healing, TGF-β and AP-1 coordinate the transient upregulation of MMP-3, which facilitates matrix degradation and remodeling. This is followed by the down-regulation of MMP-3 as tissue repair progresses and the ECM is restored. In contrast, in pathophysiological states such as fibrosis, cancer, and autoimmune diseases, dysregulated transcription factor activity leads to sustained and often excessive MMP-3 expression.

### 2.2. Post-transcriptional regulation

While transcriptional regulation governs the initiation of MMP-3 expression, post-transcriptional mechanisms play a critical role in modulating the stability and translation of MMP-3 mRNA. MicroRNAs (miRNAs), small non-coding RNAs that regulate gene expression by binding to complementary sequences in the 3′ untranslated region (3′ UTR) of target mRNAs, are key players in this process [49].

Several miRNAs have been shown to target MMP-3 mRNA, reducing its stability and inhibiting translation. For instance, miR-146a is upregulated in response to inflammatory stimuli and directly targets MMP-3 mRNA, thereby limiting its expression and preventing excessive ECM degradation [50]. This regulation is crucial in maintaining tissue homeostasis, particularly in inflammatory conditions where unchecked MMP-3 activity could lead to detrimental tissue damage. Similarly, miR-155, another inflammation-associated miRNA, has been implicated in controlling MMP-3 levels by binding to its mRNA and reducing its translation [51].In cancer, miRNAs also play an important regulatory role by modulating MMP-3 activity. By fine-tuning the expression of MMP-3 in response to various microenvironmental cues, miRNAs contribute to the balance between ECM degradation and remodeling, a key factor in tumor invasion and metastasis. Disruption in miRNA-mediated regulation can lead to aberrant MMP-3 expression, promoting pathological ECM remodeling and cancer progression [52, 53].

Rapid and cell-type-specific responses to external stimuli such as cytokines or hypoxia performed from post-transcriptional regulation, However, the biological significance of these layers of control is often inferred from limited experimental systems, indicating a need for more physiologically relevant models. Understanding how these layers integrate with transcriptional inputs will be key to fully mapping MMP-3 regulatory circuits.

### 2.3. Proteolytic activation

MMP-3 is synthesized as an inactive zymogen, proMMP-3, which requires proteolytic cleavage for activation. This tightly regulated process ensures that MMP-3 activity is restricted to sites where it is needed, preventing unnecessary ECM degradation that could lead to tissue damage.

Proteolytic activation of proMMP-3 is achieved through cleavage by other proteinases, including neutrophil elastase, plasmin, and plasma kallikrein [54,55]. These enzymes cleave the “bait” region of proMMP-3, exposing the active site and converting it into its mature, active form [56]. This activation is often part of a cascade involving multiple MMPs and other factors, like parasite cysteine proteinase A5 (CP-A5), where one MMP activates another, amplifying the proteolytic response in tissues undergoing remodeling [57]. Activated MMP-3 is capable of degrading a wide variety of ECM components, including proteoglycans, laminins, fibronectins, and collagens, particularly collagen types III, IV, and V [4]. This activity is essential for normal tissue repair processes, but excessive activation of MMP-3 can contribute to pathological conditions such as arthritis, where it degrades cartilage, or cancer, where it facilitates tumor invasion by breaking down basement membranes. Proteolytic activation of MMP-3 also plays a significant role in inflammation, where activated MMP-3 can modulate the inflammatory response by processing cytokines and chemokines.

MMP-3’s activation is tightly coordinated with local tissue remodeling events. Post-secretional regulation, such as reuptake via endocytosis or interaction with membrane proteins, further modulates its spatial activity. The cell-specific routes of MMP-3 trafficking and its non-proteolytic functions suggest that its biological role extends beyond matrix degradation. Further dissection of its secretion pathways and intracellular dynamics could offer new insights into MMP-3 regulation.

### 2.4. Inhibition by TIMPs

Once activated, MMP-3 activity is regulated by endogenous inhibitors known as tissue inhibitors of metalloproteinases (TIMPs) [58]. TIMP-1 and TIMP-2 are the primary inhibitors of MMP-3, forming stable 1:1 complexes with the active enzyme to block its catalytic activity [59]. This inhibition prevents excessive ECM degradation and helps maintain tissue integrity, particularly in processes such as wound healing and normal tissue turnover [60]. However, in pathological conditions such as cancer and fibrosis, the balance between MMP-3 and TIMP expression is often disrupted, leading to uncontrolled ECM degradation. In cancer, for instance, reduced levels of TIMP-1 and TIMP-2 have been associated with increased MMP-3 activity, facilitating tumor invasion and metastasis [61,62]. Similarly, in fibrotic diseases, where excessive ECM deposition occurs, dysregulation of TIMP-MMP interactions contributes to the progression of fibrosis by allowing unchecked matrix degradation and remodeling. In rheumatoid arthritis, increased MMP-3 activity has been linked to joint destruction, where insufficient TIMP expression fails to counterbalance the elevated levels of MMP-3. This results in the degradation of cartilage and other joint structures, exacerbating disease severity. Therapeutic strategies aimed at restoring the balance between TIMPs and MMP-3 are being explored as potential treatments for these diseases.

MMP-3 activity is finely balanced by endogenous inhibitors, mainly TIMPs. These interactions not only control the net proteolytic activity but also influence the activation of other MMPs by MMP-3. Dysregulation of the MMP-3/TIMP balance is implicated in multiple pathologies discussed below. Despite known inhibitory mechanisms, the context-specific regulation of TIMP expression and its feedback on MMP-3 activity remains an underexplored area, especially in chronic inflammatory conditions.

The regulation of MMP-3 is governed by a complex and multiple-layered network of transcriptional, translational, post translational, and inhibitory mechanisms. Each level contributes to its tightly controlled activity in physiological settings and its dysregulation in disease. While the literature offers rich data across individual regulatory axes, integrated models that unify these findings remain sparse. Future work should aim to bridge mechanistic studies with disease-relevant models to better understand how MMP-3 regulation aligns with tissue context and pathology severity.

## 3. Targeting MMP3 in diseases treatment has been an area of interest for decades

The involvement of MMP-3 in a wide range of diseases has positioned it as a significant target for therapeutic interventions. Given the extensive biological functions of MMP3 and its involvement in a variety of diseases, and more recently, with several physiological functions of MMP-3 and other MMPs family members revealed, this molecule can be applied as an indicator and therapeutic target of diseases. Due to its broad substrate spectrum and its involvement in numerous physiological and pathological processes, MMP-3 has long been recognized as a potential therapeutic target in various diseases. MMP-3’s ability to degrade ECM components, activate other proteases, and regulate cytokine activity links it to the progression of multiple disorders, including inflammatory conditions, cardiovascular diseases, neurodegenerative disorders, and cancer. The focus on inhibiting MMP-3 in these diseases stems from its role in tissue destruction, inflammation, and tumor invasion. This section will explore MMP-3’s role across several disease types and the therapeutic efforts targeting its activity. In addition to reviewing clinical and therapeutic research, this section also draws upon critical insights from Mmp3-deficient mouse models, which offer mechanistic evidence supporting MMP-3’s contribution to disease processes across different organ systems. And in more detail, we summarize and list the clinical applications of MMP-3 based on existing studies (Table 1). Targeting MMP-3 has been a topic of significant interest across a variety of disease models, as its proteolytic activity contributes to both physiological tissue remodeling and pathological processes.

### 3.1. MMP-3 involved in inflammation and Inflammatory Diseases

MMP-3 plays a pivotal role in inflammation and tissue remodeling, contributing to the pathophysiology of several inflammatory diseases. As a protease capable of degrading extracellular matrix components, MMP-3 is not only involved in tissue repair but also exacerbates inflammation in various conditions.

**Joint inflammation and arthritis** are the most common occurrence inflammatory diseases that MMP-3 engaged in. Sun et al. found that the active form of MMP-3 is a marker of synovial inflammation and cartilage degradation in joint diseases like RA. The enzyme’s role in cartilage turnover makes it a valuable biomarker for monitoring disease progression in patients with inflammatory arthritis [63]. RA, a chronic autoimmune disorder characterized by inflammation and joint destruction, and it contributes to the breakdown of cartilage and other joint structures, exacerbating the damage caused by inflammatory processes in RA. The enzyme’s activity in degrading proteoglycans and collagen makes MMP-3 a critical factor in joint damage [64–66]. Elevated levels of serum MMP-3 correlate with disease activity and joint destruction in RA patients. It serves as a biomarker to monitor disease progression and therapeutic efficacy [67–70]. Studies have shown that MMP-3 levels are significantly to be associated with clinical markers of RA activity and radiological progression, highlighting its utility as a diagnostic tool (Table 1) [71–79]. Changes in serum MMP-3 levels can reflect the therapeutic response or effectiveness of treatments such as methotrexate and biological agents like tocilizumab, with reductions in MMP-3 often correlating with clinical improvement [80–85]. In osteoarthritis (OA), MMP-3 plays a role similar to that in RA, where it degrades cartilage matrix components, leading to the joint degeneration characteristic of OA. MMP-3 is implicated in the pathogenesis of OA through its enzymatic activity on cartilage components [65,86–90]. Inhibiting MMP-3 activity is a potential strategy to protect cartilage from degradation and slow OA progression. Psoriatic Arthritis (PsA), an inflammatory arthritis to be associated with psoriasis, a chronic skin and nail disease is similar to RA in symptoms and joint swelling (inflammation), which also shows involvement of MMP-3 in joint inflammation, damage and therapeutic potential. Elevated MMP-3 levels were found to be associated with increased joint damage and inflammation in PsA patients, which also involved other biological molecules in the PsA pathogenesis like myeloperoxidase (MPO), MMP-9, CD44, IL15 and TWEAK etc [91–94]. MMP-3 together with other biological molecules could serve as biomarkers for PsA. MMP-3-deficient mice still develop arthritis in CIA models, though cartilage degradation varies by disease stage. Mudgett et al. (1998) showed that MMP-3 is not essential for disease onset but influences the extent and pattern of cartilage damage [97]. This suggests partial redundancy among MMPs, with MMP-3 contributing to—but not solely driving—matrix breakdown. Thus, its inhibition may reshape rather than fully prevent disease progression.

Given the strong correlation between elevated MMP-3 levels and inflammatory joint diseases, it is important to further explore the specific mechanisms driving MMP-3 expression and activity in distinct clinical entities such as RA and PsA. In RA, MMP-3 is upregulated by inflammatory cytokines like TNF-α and IL-1β, driving synovial inflammation and cartilage degradation. Elevated MMP-3 levels contribute to the breakdown of matrix components, exacerbating joint damage. Its activity correlates with disease severity, where increased MMP-3 worsens both tissue destruction and inflammation by releasing cytokines such as TGF-β and EGF, which further perpetuate the inflammatory cycle. In PsA, MMP-3 similarly contributes to joint inflammation and damage. Elevated MMP-3 is linked to increased disease activity, joint swelling, and tissue invasion by inflammatory cells. Studies suggest that MMP-3, in combination with other markers like MMP-9 and CD44, may serve as valuable biomarkers for monitoring disease progression in PsA. Therapeutically, reducing MMP-3 levels using biologics like TNF inhibitors has shown promise in improving disease activity and preserving joint integrity in both RA and PsA. Thus, MMP-3 remains a critical target for intervention to limit both inflammation and structural damage in these diseases.

**Obesity**, characterized by excessive adipose tissue accumulation, is to be associated with chronic low-grade inflammation, and MMP-3 is increasingly recognized as a key player in these processes, influencing adipose tissue expansion and metabolic function. Wu et al. demonstrated that obesity induced by a high-fat diet increases MMP-3 expression in a depot- and sex-dependent manner in mouse model, suggesting that MMP-3 plays a role in the differential metabolic responses to obesity across various fat depots [95]. Similarly, Boumiza et al. investigated the Lys45Glu variant of MMP-3, which alters MMP-3 activity and is associated with microvascular reactivity and obesity phenotypes in a Tunisian population, suggesting a genetic contribution of MMP-3 to obesity complications [96]. The link between obesity and inflammation is further emphasized in the study by Williams et al., which showed that leptin—a hormone elevated in obesity—synergistically increases MMP-3 secretion when combined with pro-inflammatory stimuli. This process, observed in human gingival fibroblasts, suggests that MMP-3 is part of the inflammatory cascade triggered by obesity and may contribute to related comorbidities such as periodontal disease [97]. In humans, the association between MMP-3 and visceral adipose tissue activity has also been explored. Serretta et al. examined biochemical markers of visceral adipose tissue, including MMP-3, and their correlation with body mass index (BMI) and visceral adiposity index (VAI). Their findings suggest that MMP-3, along with other markers like leptin and adiponectin, positively correlated with visceral fat activity and can serve as an indicator of obesity severity in clinical settings [98]. Interestingly, MMP-3 appears to modulate tissue growth under metabolic stimulation in MMP-3 knockout mice models. Maquoi et al. [99] demonstrated enhanced adipose tissue development in MMP-3 knockout mice under a high-fat diet, indicating that MMP-3 constrains adipose expansion, likely via matrix remodeling mechanisms. This observation contrasts with human studies, where MMP-3 levels are elevated due to chronic inflammation and its role in ECM remodeling during obesity. In the MMP-3 knockout mice, the absence of MMP-3 removes its contribution to the inflammation-induced upregulation of ECM remodeling, thereby limiting the downstream effects that are typically triggered by MMP-3 activity in an inflammatory environment. This suggests that in MMP-3 knockout models, the lack of MMP-3 prevents the full cascade of regulatory events that would normally facilitate tissue remodeling in response to metabolic stress. Additionally, the role of MMP-3 in preadipocyte function and fat tissue regulation was demonstrated by Traurig et al., who found that MMP-3 expression differs significantly between preadipocytes from obese and non-obese individuals. In particular, stromal vascular cells from obese nondiabetic Pima Indians exhibited elevated MMP-3 expression, indicating its potential role in adipose tissue dysfunction and expansion in obesity [100]. In summary, MMP-3 is intricately involved in the regulation of adipose tissue, inflammation, and the metabolic consequences of obesity. Its role as a mediator between adipose tissue expansion and inflammatory responses suggests that MMP-3 could be a therapeutic target for managing obesity-related complications. Although MMP-3 is generally associated with promoting obesity-related inflammation and tissue remodeling in humans, studies involving MMP-3 knockout mice present a contrasting scenario. This underscores the multifaceted and complex regulatory roles of MMP-3 in metabolic processes, highlighting the need for further research—particularly in pathological conditions—to fully elucidate its potential as both a diagnostic and therapeutic target.

#### Other inflammatory diseases:

Walker and Rosenberg highlighted the role of MMP-3 in delayed inflammation and neuronal death in the hippocampus following ischemia. Their study suggested that MMP-3, alongside TIMP-3, contributes to inflammation-induced neuronal damage, linking the enzyme to neuroinflammatory processes [101]. And MMP-3 related neurodegenerative diseases have been discussed below. In dental tissue, Goda et al. explored how TNF-α enhances MMP-3 production in human dental pulp fibroblast-like cells. This highlights MMP-3’s role in oral inflammation, linking it to broader inflammatory pathways activated by TNF-α [102]. Van Hove et al. investigated the role of MMP-3 in endotoxin-induced inflammation in the posterior eye segment. Their findings showed that MMP-3 deficiency alleviated acute inflammation, underscoring its potential as a therapeutic target in inflammatory eye diseases [103]. Zhang et al. demonstrated that MMP-3 plays a crucial role in pulmonary inflammation and fibrosis induced by copper oxide nanoparticles. This highlights the enzyme’s involvement in toxic environmental exposures, leading to significant inflammatory responses in the respiratory system [104]. Roy et al. demonstrated that inducible MMP-3 expression in human microvascular endothelial cells is modulated by anti-inflammatory agents, indicating its role in regulating vascular responses during inflammation. This suggests that MMP-3 is involved not only in local tissue inflammation but also in systemic vascular responses [105]. **Systemic Lupus Erythematosus (SLE),** a systemic autoimmune disease affecting multiple organs. MMP-3 contributes to the degradation of tissue structures, exacerbating the inflammatory responses in SLE. Elevated MMP-3 levels are linked to increased tissue damage and inflammation in SLE patients including the patients with lupus nephritis [106–108]. Modulating MMP-3 activity could be beneficial in reducing systemic inflammation and tissue damage in SLE patients.

Collectively, MMP-3 plays a pivotal role in the pathophysiology of several inflammatory diseases by contributing not only to inflammatory cascades but also to ECM degradation and tissue remodeling. Its proteolytic activity targets a wide array of ECM components, including collagens, fibronectin, and proteoglycans, leading to the structural disintegration of tissue architecture. This ECM breakdown not only facilitates leukocyte infiltration and propagation of inflammation, but also alters mechanical and biochemical properties of the tissue, contributing to chronic tissue damage and impaired regeneration. In diseases such as rheumatoid arthritis, PsA, and TNF-α active inflammation diseases, such MMP-3–mediated remodeling amplifies disease progression by disrupting tissue homeostasis and underscore the dual pro-inflammatory and tissue-remodeling roles of MMP-3 in disease progression.

### 3.2. Role of MMP-3 in infectious and infectious diseases

In recent years, it has garnered attention for MMP-3’s involvement in the pathology of various infectious diseases, including viral, bacterial, and fungal infections. One of the most discussed roles of MMP-3 is in the context of Coronavirus disease (COVID-19). Studies have shown that MMP-3 contributes to the inflammatory responses seen in COVID-19 patients, with differential effects in adults and children. Toczyłowski et al. demonstrated significant variations in inflammatory markers, including MMP-3, between adult and pediatric COVID-19 patients, suggesting potential long-term consequences and the need for anti-inflammatory treatments in severe cases [109]. Similarly, Ramezani et al. highlighted the association of MMP family gene polymorphisms, including MMP-3, with the risk of developing neurological symptoms in COVID-19 patients, reinforcing the systemic impact of MMP-3 in viral infections [110]. Gelzo et al. identified MMP-3, alongside MMP-9, as a biomarker of disease severity in COVID-19 patients, indicating its potential as a prognostic tool for identifying high-risk individuals [111]. Almuntashiri et al. further explored MMP-3 as a potential therapeutic target in severe COVID-19, emphasizing its role in exacerbating inflammation and tissue damage in the lungs. The therapeutic inhibition of MMP-3 has been proposed as a possible intervention for managing acute respiratory distress syndrome (ARDS) in COVID-19 patients, as discussed by Kadry et al. [112,113]. Shi et al. also supported this view, suggesting MMP-3 as a valuable marker for disease progression in COVID-19 [114]. Beyond COVID-19, MMP-3 has been implicated in broader infectious diseases. Lee and Kim reviewed the role of MMP-3 in inflammation across a variety of infectious diseases, noting its involvement in tissue remodeling and immune responses during infections [115]. In osteoarthritis patients recovering from COVID-19, Tuharov et al. observed elevated plasma levels of MMP-3, indicating a possible link between MMP-3 levels and post-infectious inflammatory complications [116].

MMP-3’s role is not limited to viral infections. Supasorn et al. demonstrated that MMP-3 contributes to pulmonary inflammation during Cryptococcus infections, suggesting a regulatory role in chemokine expression during fungal infections [117]. In bacterial infections, Vanlaere and Libert identified MMP-3 as a potential drug target for managing infections caused by gram-negative bacteria and septic shock, highlighting its critical involvement in infection-induced tissue damage [118]. Similarly, Van den Steen et al. explored the role of MMP-3 in cerebral malaria, showing how its activity exacerbates inflammation in severe cases of the disease [119].

Beyond its established role in chronic inflammatory diseases, MMP-3 has also been implicated in host responses to acute infections. While it is elevated in severe COVID-19 and serves as a potential biomarker for disease severity, its function in bacterial and fungal infections is increasingly recognized. In models of bacterial sepsis and endotoxin-induced inflammation, MMP-3 contributes to tissue damage by promoting leukocyte infiltration and cytokine release through ECM breakdown. Interestingly, MMP-3 exhibit increased tissue pathology and attenuated inflammatory cytokine responses in certain animal models of bacterial infection, such as lipopolysaccharide (LPS)-induced lung injury, suggesting that MMP-3 may amplify immunopathology rather than being essential for pathogen clearance. Conversely, in some contexts, MMP-3 is involved in modulating leukocyte trafficking and tissue remodeling, indicating its complex role in balancing host defense and inflammation. These findings highlight MMP-3 not only as a potential therapeutic target to control infection-associated tissue damage, but also as a prognostic biomarker for infection severity.

### 3.3. MMP-3 impacts on cardiovascular diseases

In addition to its role in inflammatory and infection diseases, MMP-3 also plays a significant role in cardiovascular diseases (CVDs), particularly in the development and progression of coronary artery disease (CAD), which has been the focus of numerous studies. One of them is genetic polymorphisms and CAD susceptibility. Humphries argued that MMP-3 has “won the election” as a strong candidate gene for CAD, given the accumulating evidence of its genetic variations and their clinical consequences [120]. Early research by Ye also highlighted the influence of MMP genotypes on cardiovascular outcomes, suggests that understanding these genetic factors could improve patient risk stratification and treatment [121]. Li’s meta-analysis demonstrated a significant link between MMP3 polymorphisms and CAD susceptibility, especially with variants in the promoter region of the gene, such as the 5 A/6 A polymorphism [122]. Similarly, Shalia et al. found a higher prevalence of the 5 A/6 A promoter polymorphism in CAD patients in an Indian population, suggests this polymorphism may increase the risk of CAD [123]. However, conflicting findings exist, as McGlinchey et al. reported no association between the 5 A/6 A polymorphism and ischemic heart disease in a family-based study, highlighting the complexity of genetic influences [124]. In addition to genetic predisposition, circulating levels of MMP-3 have been proposed as biomarkers for cardiovascular outcomes. Wu et al. demonstrated that plasma MMP-3 levels was an independent prognostic factor in patients with stable CAD, correlating with future cardiovascular events such as myocardial infarction and death [125]. This was further supported by Guizani et al., whose cohort study over five years identified MMP-3 as a predictive marker for clinical cardiovascular outcomes in CAD patients [126]. Furthermore, MMP-3 does not act in isolation but interacts with other proteases and inflammatory mediators in cardiovascular pathology. Guizani et al. emphasized the interplay between MMP-3 and MMP-9 in their cohort study, suggests that both proteases serve as genetic biomarkers for cardiovascular complications in CAD patients [127]. Moreover, Krumsiek et al. examined the combined influence of MMP-3 with other genetic factors, such as TNF-α polymorphisms, and found that these interactions may modulate the extent of ischemic heart disease [128]. In addition to the studies mentioned above, the role of MMP-3 in CAD has also been explored in various ethnic populations. Ghaffarzadeh et al. studied Iranian Turks and found a significant association between the MMP3 (rs3025058) 6 A/6 A genotype and CAD risk [129]. Similarly, Beton et al. reported that polymorphisms in both MMP-3 and MMP-9 was associated with CAD in Turkish patients [130]. Furthermore, the involvement of MMP-3 extends beyond traditional CVDs. Mohammadhosayni et al. investigated the role of MMPs in the neurological complications of COVID-19 and found that MMP-3 contributed to the vascular and inflammatory damage seen in patients with severe cases, suggests its broader role in systemic vascular diseases [131]. The clinical relevance of MMP-3 in cardiovascular research suggests that it is a candidate gene of interest in CVD studies. In post-myocardial infarction models, MMP-3 has been linked to adverse outcomes such as cardiac rupture. MMP-3 inhibition or genetic ablation prevented rupture but also impaired reparative angiogenesis, suggesting a dual role in cardiac ECM remodeling [132]. This exemplifies how MMP-3 functions can be both deleterious and reparative, depending on the tissue and phase of injury.

In cardiovascular pathologies, MMP-3 emerges as both a contributor to tissue damage and a measurable marker of disease progression. Polymorphisms like the 5 A/6 A variant influence individual susceptibility to coronary artery disease. Meanwhile, elevated circulating MMP-3 levels correlate with adverse outcomes (such as myocardial infarction and mortality) in coronary artery disease patients, positioning this protease as an independent prognostic indicator in clinical setting. Mechanistically, MMP-3 drives atherosclerotic plaque instability and vascular remodeling by degrading extracellular matrix components and activating other proteases, thereby weakening fibrous caps and promoting inflammation within lesions. Beyond coronary disease, MMP-3’s influence extends to broader cardiovascular conditions, where its expression is associated with myocardial remodeling, endothelial dysfunction, and post-injury inflammation. Collectively, these findings underscore MMP-3’s multifaceted role in cardiovascular disease – its genetic variants, enzymatic activity, and crosstalk with inflammatory mediators all contribute to disease development and progression – making MMP-3 both a valuable biomarker of cardiovascular risk and a potential therapeutic target for preventing adverse cardiac events.

### 3.4. MMP-3 in neurodegenerative diseases

Structural and functional synapse reorganization is one of the key issues of learning and memory mechanisms. MMP-3 has been identified as a major brain proteases affecting numerous processes and plays a pivotal role during learning-related modification of neural circuits. In the nervous system, the typical pathological changes are extensive inflammation and axonal injury. MMP-3 can degrade the ECM proteins in the brain, leading to the destruction of neurons and synaptic loss. And excessive uncontrolled activity of MMP-3 often correlates with the disruption of the blood–brain barrier (BBB), role in demyelination, cell apoptosis or initiation of the inflammatory response that implicated MMP-3 play roles in the pathogenesis of neurodegenerative diseases such as Alzheimer’s disease (AD) and Parkinson’s disease (PD) [133, 134]. Other central nervous system degenerative diseases, such as multiple sclerosis, have also been investigated for the role of MMP-3 in modulating B lymphocyte aggregates [135].

MMP3 in Alzheimer’s disease: The main characteristic features of AD are a widespread degeneration of neurons. The formation of so-called plaques that contain amyloid-β (Aβ) peptide, the product of proteolytic cleavage of amyloid precursor protein (APP), and deposition of neurofibrillary tangles abnormally rich in hyperphosphorylated tau protein. And it is feasible to use of MMP-3, other MMPs/TIMPs as well as growth factors as markers of differences and progression in AD [136–141]. Zhang and colleagues found a close correlation between P-tau (S199) and MMP-2/MMP-3 in Alzheimer’s disease (AD) and hearing loss (HL) patients CSF [142]. Though the mechanism of MMP-3 contributes to the development of AD is complex, the expression level of MMP-3 is significantly correlated with the level of Aβ in the human cerebrospinal fluid [143]. And the correlation between MMP3 and AD is also reflected in the fact that the expression level of MMP-3 is positively to be associated with the progression of AD, especially related to the gene morphisms of MMP-3 [144,145]. As a biomarker for diagnosis of AD, MMP-3 has been extensively investigated in clinics (Table 1).

MMP3 in Parkinson’s disease: The involvement of MMP-3 together with other MMPs and regulators in the pathophysiology of Parkinson’s Disease (PD) may be linked to its role in activating microglia and triggering inflammatory responses due to cellular stress [133,146,147].The activation of MMP-3 from pro-MMP-3 in the cytosol under a cell stress condition might due to the translocation of serine protease HtrA2/Omi from mitochondrial to cytosol, which can ultimately lead to demise of dopaminergic neuronal cells [148]. Additionally, alterations in BBB permeability have been observed in both PD patients and animal models of the disease [149]. MMP-3 is known to target tight junction proteins, suggesting that MMP-3-induced BBB permeability changes are a potential mechanism contributing to PD. Proteins to be associated with PD mutations are also substrates for MMP-3. Notably, α-synuclein, a key component of Lewy bodies and a significant factor in PD development, is cleaved by MMP-3. This cleavage exposes a hydrophobic region on α-synuclein, promoting the formation of protein aggregates. Furthermore, mutations in α-synuclein linked to early-onset PD increase its presence in Lewy bodies, implying that the mutant protein may be more susceptible to MMP-3-mediated degradation. Another facet of MMP-3 involvement in PD pathogenesis pertains to its interaction with protein DJ-1, which plays a protective role against oxidative, proteasomal, and mitochondrial stress. Mutations and a loss of functional activity in DJ-1 have been documented in PD patients. Given that DJ-1 is a substrate for MMP-3, it is plausible that the diminished activity of DJ-1 observed in PD could be attributed to the heightened, uncontrolled proteolytic activity of MMP-3 [150]. In conclusion, the role of MMP-3 in the pathophysiology of PD is manifested through the contribution of protease to the formation of α-synuclein aggregates and by the interference of the MMP-3 protease with the protective functions of DJ-1 protein against oxidative stress. Overall, MMP-3’s role in PD pathogenesis involves its contribution to the formation of α-synuclein aggregates and its impact on cellular protective mechanisms. Additionally, evidence from animal models supports the structural role of MMP-3 in the central nervous system. For instance, Aerts et al. [151] showed that MMP-3-deficient mice exhibited altered neuronal plasticity in the adult visual cortex, further suggesting that MMP-3 contributes to maintaining neural architecture and synaptic remodeling under physiological conditions.

Collectively, these findings underscore MMP-3 as a multifunctional protease involved in the onset and progression of several neurodegenerative disorders. Its roles span from ECM remodeling and BBB disruption to modulation of neuroinflammatory signaling and degradation of neuroprotective proteins. The context-specific activity of MMP-3, particularly in AD and PD, highlights its potential not only as a biomarker but also as a therapeutic target for limiting neuronal damage and disease progression.

### 3.5. Role of MMP3 in cancer

Regarding its role in malignancy, research has indicated that MMP-3 exhibits both tumor-promoting and tumor-inhibiting properties, contingent upon the substrates with which it interacts [152]. For example, MMP-3 catalyzes the formation of angiogenesis-inhibiting factors through the degradation of plasminogen and type VIII collagen [153,154]. These factors restrict tumor progression, thereby demonstrating MMP-3’s tumor-suppressive properties in such contexts. Conversely, when modulating growth factors, such as transforming growth factor, MMP-3 stimulates the proliferation of cancer cells and tumor progression [152,155]. Despite these dual functions, MMP-3 is more frequently implicated in promoting tumor development rather than suppressing it [152,156].

MMP-3 is regarded as a prognostic marker in various types of cancers. Breast cancer is one of the most widely explored cancer regarding the MMP-3 roles involved in from genotype to protein expression levels [7,153,157–159]. Camacho et al. [160]. reported elevated expression levels of MMP-3 in breast cancer tissue compared to normal breast tissue, and Lawicki et al. [161] reported the plasma Levels of MMP-3 together with MMP-7 and CA 15–3 can be used as new tumor biomarkers in the diagnosis of breast cancer, especially in Her-2 negative cancer, suggests a potential association with breast cancer development. Similarly, upregulated expression of MMP-3 was also observed in oral squamous cell carcinoma, the most common form of oral cancer, which can be useful in diagnosis of squamous cell carcinoma [48,162, 163]. Furthermore, recent studies imply that MMP-3 plays a role in the progression of prostate cancer to bone metastasis and upregulated in both prostate cancer cells and cancer-to be associated stromal fibroblasts (CAFs) [29,41,164]. Additionally, relationship of single nucleotide polymorphisms (SNPs) in functional domain of MMP-3 in bladder cancer has been investigated, which contribute appreciably to cancer predisposition and aggression [165,166]. Recent reports exhibited that both protein levels and gene variation of MMP-3 and other MMPs (e.g. MMP-1, MMP-9) are to be associated with high risk of bladder cancer and pancreatic cancer [9,167,168]. Chen et al. have elucidated the critical involvement of ubiquitin-specific peptidase 15 and MMP-3 in the development and prognosis of non-small-cell lung cancer [169].Cymbaluk-Ploska et al. elucidated a negative correlation between expression level of MMP-3 and TIMP-3 in ovarian cancer, which means higher level of MMP-3 exhibited shorter patients’ survival [170]. Therefore, MMP-3 represents a promising target for the development of novel antineo-plastic drugs and diagnosis biomarker.

In cancer, MMP-3 exhibits dual roles. On one hand, MMP-3 can limit tumor growth by generating angiogenesis inhibitors. On the other hand, MMP-3 more commonly facilitates malignancy by remodeling the tumor microenvironment and modulating signaling molecules: it degrades basement membranes to ease cancer cell invasion and activates growth factors that stimulate tumor cell proliferation and metastatic progression. In numerous malignancies (e.g. breast, oral, prostate, bladder, and lung cancers), MMP-3 is frequently overexpressed, and this upregulation correlates with more aggressive disease features and poorer patient prognose. These clinical associations have established MMP-3 as a promising prognostic biomarker across cancer types, useful for indicating tumor burden or likelihood of metastasis. Consequently, these findings support its use as a prognostic biomarker and therapeutic target, with emerging strategies aiming to block MMP-3’s pathological signaling while sparing its physiological roles.

### 3.6. MMP-3’s roles in organ-specific fibrotic diseases

Given the broad involvement of MMP-3 in various pathological conditions, it is particularly important to understand both its shared mechanisms across fibrotic diseases and the organ- specific contexts that modulate its activity. Across different organs, MMP-3 has been implicated in fibrotic diseases through both shared and organ-specific mechanisms. Pulmonary fibrosis: MMP-3 is markedly upregulated in the lungs of idiopathic pulmonary fibrosis patients and animal models, where it drives myofibroblast accumulation and aberrant tissue remodeling by activating pro-fibrotic pathways such as Wnt/β-catenin signaling and converting latent TGF-β_1_ to its active form [171]. In pulmonary fibrosis models, MMP-3 has been shown to mediate Epithelial–mesenchymal transition (EMT) and enhance matrix remodeling. Mmp3 knockout mice subjected to lung injury demonstrated reduced collagen deposition and decreased inflammation, indicating a contributory role of MMP-3 in driving fibrogenesis through ECM degradation and fibroblast activation [172] (Puntorieri et al., 2016). Similarly, MMP-3-deficient mice show altered skin remodeling and reduced TNF-α-induced collagen breakdown (Mirastschijski et al., 2019), emphasizing its key function in ECM turnover in fibrotic contexts. Hepatic fibrosis: In chronic liver disease (e.g. biliary cirrhosis), elevated MMP-3 levels correlate with advanced fibrosis and liver dysfunction, suggesting that MMP-3 contributes to hepatic stellate cell activation and matrix deposition during cirrhosis [173]. Renal fibrosis: MMP-3 is also linked to chronic kidney disease progression – high MMP-3 levels are associated with worse renal function, consistent with MMP-3 promoting tubular epithelial–mesenchymal transition and interstitial fibrosis in the kidneypubmed.ncbi.nlm.nih.gov [174]. Pancreatic fibrosis: Activated pancreatic stellate cells and periacinar myofibroblasts in chronic pancreatitis secrete MMP-3, which degrades ECM and facilitates sustained inflammation, thereby contributing to the fibrotic replacement of pancreatic tissuejournals.lww.com [175]. Shared among these conditions is MMP-3’s ability to remodel the extracellular matrix and release pro-fibrotic factors (like active TGF-β), thereby perpetuating fibrosis. However, the predominant cell sources and downstream effects of MMP-3 can differ by organ – for instance, alveolar epithelial cells and macrophages are major sources in the lung, whereas hepatic and pancreatic stellate cells play a central role in liver and pancreas.

Despite variations in specific regulatory pathways and organ contexts, a consistent mechanism across pulmonary, hepatic, renal, and pancreatic fibrosis involves MMP-3-driven ECM remodeling. In these fibrotic conditions, elevated MMP-3 expression uniformly promotes degradation and turnover of ECM components such as collagen, fibronectin, and proteoglycans. This excessive and persistent ECM remodeling contributes to pathological tissue restructuring and fibrosis progression by disrupting normal tissue architecture, releasing active pro-fibrotic growth factors like TGF-β, and facilitating the recruitment and activation of fibrogenic cells (e.g., myofibroblasts and stellate cells). Recognizing this common ECM remodeling activity of MMP-3 provides a fundamental rationale for targeting MMP-3 as a therapeutic strategy across diverse fibrotic disorders.

MMP-3 plays a unifying role across diseases by driving ECM degradation and amplifying inflammation. In inflammatory and infectious conditions, MMP-3 contributes to tissue destruction and reflects disease activity. In cardiovascular and neurodegenerative disorders, it destabilizes structural barriers like plaques and the blood–brain barrier. In cancer and fibrosis, it remodels the microenvironment to support invasion or fibrotic accumulation. Emerging research is now focused on context-specific modulation of MMP-3, aiming to selectively inhibit its deleterious effects (for example, using MMP-3 inhibitors to stabilize vulnerable plaques or attenuate post-injury fibrosis) while preserving its normal physiological functions in tissue maintenance. Such precision strategies underscore the translational potential of MMP-3, as efforts to harness or inhibit this protease could yield novel diagnostics and treatments that cut across traditional disease boundaries.

## 4. Molecular pathways mediated by MMP3

**Fig. 2.**
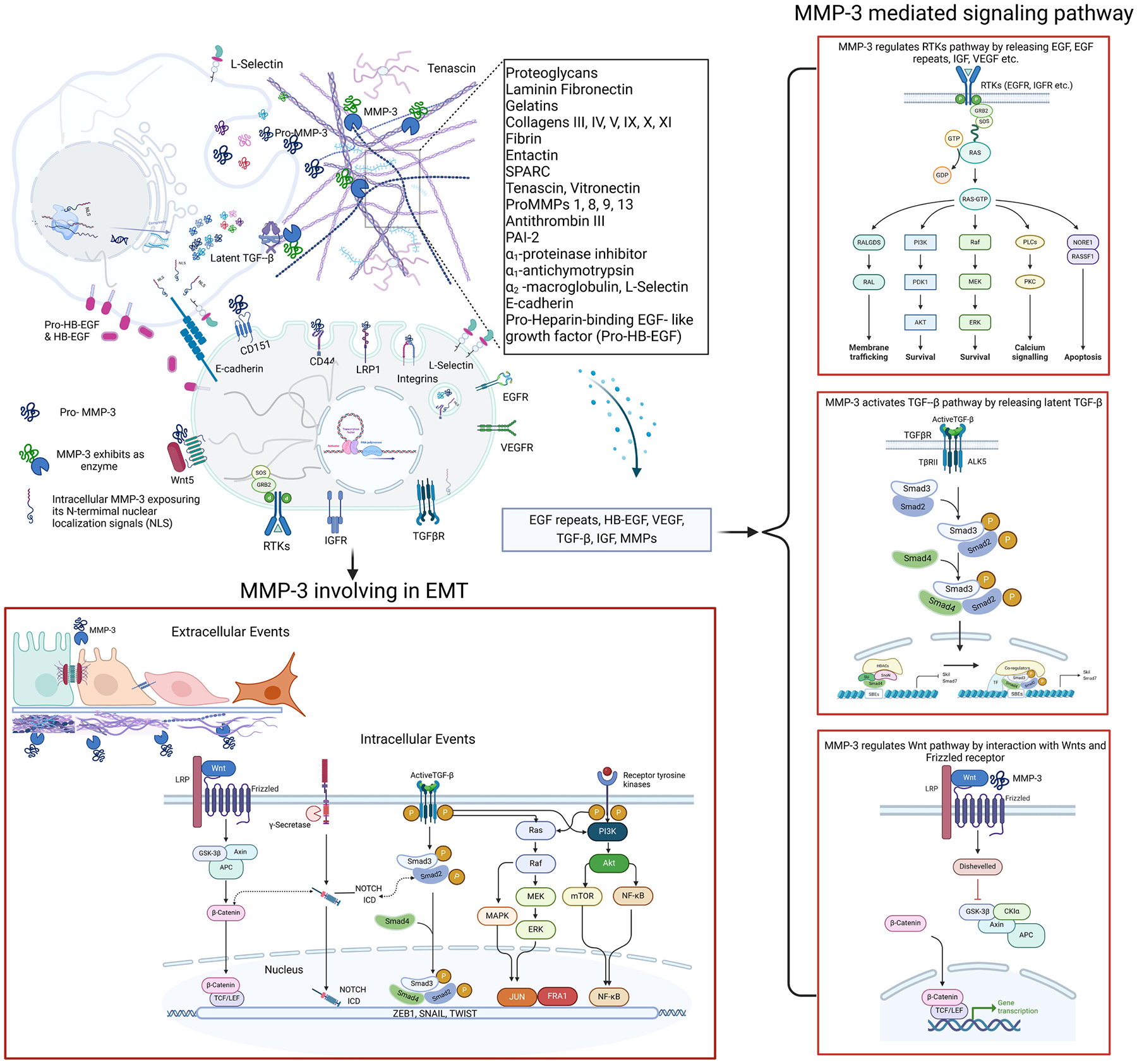
The role of MMP-3 in physiological and pathological biological processes involving both ECM-dependent and ECM-independent molecular pathways. As an ECM-degrading enzyme, MMP-3 acts as a versatile regulator of cellular signaling by interacting with various substrates, including proteoglycans, laminin, fibronectin, gelatin, collagens (III, IV, V, IX, X, XI), link protein, fibrin, entactin, SPARC, tenascin, vitronectin, ProMMPs (1, 8, 9, 13), antithrombin III, PAI-2, α1-proteinase inhibitor, α1-antichymotrypsin, α2-macroglobulin, L-selectin, E-cadherin, and heparin-binding EGFlike growth factor (HB-EGF), among others. Through its proteolytic activity, MMP-3 activates various signaling pathways, particularly RTK-related pathways by releasing factors such as EGF, EGF repeats, IGF, and VEGF, as well as the TGF-β pathway. MMP-3 also influences signaling directly by modulating the availability of ligands and receptors, including Wnt family molecules and their Frizzled receptors. Beyond its extracellular roles, MMP-3 has been shown to participate in transcriptional regulation through intracellular signaling via its nuclear localization signals (NLS).Abbreviations: ECM = extracellular matrix; SPARC = secreted protein acidic and rich in cysteine; ProMMPs = pro-matrix metalloproteinases; EGF = epithelial growth factor; Pro-HB-EGF = pro-heparin-binding EGF-like growth factor; PAI-2 = plasminogen activator inhibitor-2; RTK = receptor tyrosine kinase; RTKRs = RTK receptors; IGF = insulin-like growth factor; IGFR = IGF receptor; VEGF = vascular endothelial growth factor; VEGFR = VEGF receptor; LRP1 = low density lipoprotein receptor-related protein 1; EMT = epithelial-mesenchymal transition; GSK3β = glycogen synthase kinase-3 beta; APC = adenomatous polyposis coli; TCF/LEF = T cell factor/lymphoid enhancer factor; ICD = intracellular domain; ZEB1 = zinc finger E-box-binding homeobox 1; SNAIL = zinc finger protein SNAI1; TWIST = Twist-related protein also known as class A basic helix–loop–helix protein; RALGDS = Ral guanine nucleotide dissociation stimulator; RAL = ras-related protein; PLC = phospholipase C; PKC = protein kinase C; NORE1 = novel Ras/Rap effector 1, also called RASSF5; RASSF1 = Ras association domain-containing protein 1; ALK5 =activin receptor-like kinase 5, also known as TβRI; TβRII = TGF-β receptor II; TF = transcription factor; SBEs = Smad binding element.

Matrix metalloproteinase-3 (MMP-3) mediates a broad spectrum of biological processes by engaging in both ECM-dependent and ECM-independent molecular pathways. Initially characterized as an ECM-degrading enzyme, MMP-3 has emerged as a versatile regulator of cellular signaling through its interactions with various substrates and signaling molecules, which include proteoglycans, laminin, fibronectin, gelatins, collagens III, IV, V, IX, X, XI, link protein, fibrin, entactin, SPARC, tenascin, vitronectin, proMMPs 1, 8, 9, and 13, antithrombin III, PAI-2, α_1_-proteinase inhibitor, α_1_-antichymotrypsin, α_2_ -macroglobulin, L-selectin, E-cadherin, and heparin-binding EGF- like growth factor (HB-EGF) etc [4]. This dual functionality allows MMP-3 to influence processes ranging from tissue remodeling and inflammation to cell proliferation, differentiation, and apoptosis. The enzyme’s involvement in multiple signaling cascades highlights its central role in regulating cellular responses to both physiological and pathological stimuli. The diverse molecular pathways involving MMP-3 are summarized in Fig. 2.

### 4.1. MMP-3-mediated cell signaling pathways

MMP-3’s role extends beyond degrading ECM components such as collagen, elastin, and proteoglycans. Through its proteolytic activity, MMP-3 also processes and activates a variety of signaling molecules, which in turn influence key cellular processes. One of the primary mechanisms by which MMP-3 exerts its effects is through the release of growth factors sequestered in the ECM) [176]. For example, MMP-3 can cleave and release heparin-binding epidermal growth factor (HB-EGF), a potent activator of the epidermal growth factor receptor (EGFR) pathway [177]. Upon activation, EGFR triggers downstream signaling cascades, such as the Ras/Raf/MEK/ERK (extracellular signal-regulated kinase) pathway, which regulates cell proliferation, survival, and migration [178]. By modulating the bioavailability of HB-EGF, MMP-3 indirectly contributes to the regulation of these fundamental cellular processes. This mechanism is particularly relevant in cancer, where aberrant activation of EGFR signaling drives tumor growth and metastasis.

Another important pathway influenced by MMP-3 is the **TGF-β signaling pathway**. MMP-3 can activate latent TGF-β bound to the ECM by proteolytically removing its latency-associated peptide (LAP) [179, 180]. Once released, active TGF-β binds to its receptors, triggering the SMAD-dependent signaling cascade, which controls processes like cell differentiation, fibrosis, and immune regulation [181–183]. The activation of TGF-β by MMP-3 is critical in fibrosis and cancer, where TGF-β promotes tissue remodeling and tumor progression [179].

In addition to the EGFR and TGF-β pathways, MMP-3 also participates in modulating the **Wnt signaling pathway**. Wnt signaling is crucial for regulating cell fate, proliferation, and migration, particularly in developmental biology and cancer [184,185]. MMP-3 has been shown to influence Wnt signaling by modulating the availability of Wnt ligands and receptors. For example, the non-catalytic hemopexin (HPX) domain of MMP-3 can bind to Wnt5b [186], a non-canonical Wnt ligand, affecting its interaction with the Frizzled receptors on the cell surface e [187,188]. Besides MMP-3 can transcriptionally upregulated by Wnt signaling, thereby adding MMP-3 to the list of β-catenin-regulated MMPs, upregulation of MMP-3 upon activation of Wnt signaling has also been demonstrated by Blavier’s work by using Wnt1 overexpression mammary epithelial cells [189]. This interaction promotes canonical Wnt/β-catenin signaling, which is crucial for maintaining tissue homeostasis and stem cell regulation [190].

Through these interactions, MMP-3 acts as a molecular switch, controlling the balance between different signaling pathways to maintain cellular equilibrium or, conversely, driving pathological processes when dysregulated.

### 4.2. Role of MMP-3 in epithelial-mesenchymal transition (EMT)

EMT is a cellular program in which epithelial cells lose their cell-cell junctions and apical-basal polarity, adopting a mesenchymal phenotype characterized by enhanced migratory and invasive capabilities [191, 192]. Activation of EMT results in the loss of cell polarity, disruption of cell–cell junctions, degradation of the underlying basement membrane and reorganization of the ECM [193], and it is a crucial process during embryogenesis, wound healing, and cancer metastasis. Among these pathways, TGF-β, Wnts, Notch and mitogenic growth factors engaged signaling transduction impinge on each other to collaboratively activate the EMT program [194–196]. MMP-3 plays a pivotal role in regulating EMT through its proteolytic activity and interactions with signaling pathways such as TGF-β, Wnt, Notch and receptor tyrosine kinases (RTKs).

MMP-3 is known to induce EMT by degrading E-cadherin, an adhesion molecule critical for maintaining the epithelial phenotype. The loss of E-cadherin destabilizes cell-cell junctions and facilitates the transition to a mesenchymal state. In addition to direct cleavage of E-cadherin, MMP-3 upregulates the expression of mesenchymal markers such as vimentin, further driving the EMT process [197,198]. Moreover, MMP-3 can modulate intracellular signaling pathways that regulate EMT. For example, it enhances the activity of Rac1b, a splice variant of the small GTPase Rac1, which is involved in cytoskeletal reorganization and cellular migration. The mechanism of MMP-3 upregulation Rac1b was elucidated by identification of the heterogeneous nuclear ribonucleo-protein (hnRNP) A1 as a factor involved in Rac1 splicing regulation by interaction with the splicing factors with their target sequence in pre-mRNA. When hnRNP A1 binds to the alternative exon 3b of Rac1 and has a strong repressor activity on this exon inclusion. While MMP-3 treatment alleviates Rac1 pre-mRNA exon 3b from splicing inhibition by the disassembly of a repressor complex composed of hnRNP A1, leading to an enhanced inclusion of this oncogenic exon [199]. Rac1b promotes EMT by generating ROS and activating transcription factors like Snail, which represses E-cadherin transcription and promotes mesenchymal gene expression [200]. The ability of MMP-3 to induce Rac1b expression suggests a novel mechanism by which MMP-3 drives EMT, particularly in cancer.

### 4.3. MMP-3 in other signaling pathways

In addition to the well-characterized roles of MMP-3 in the TGF-β, Wnt, and EGFR pathways, the enzyme also influences other critical signaling pathways that regulate cellular behavior. For instance, MMP-3 can modulate the **insulin-like growth factor (IGF) signaling pathway**, which is essential for cell growth, differentiation, and survival [201]. By degrading ECM components, MMP-3 releases IGF from its binding proteins, making it available to bind to the IGF-1 receptor (IGF-1R). Activation of IGF-1R triggers the PI3K/Akt pathway, which promotes cell survival and proliferation while inhibiting apoptosis. This mechanism is particularly relevant in cancer, where IGF signaling contributes to tumor growth and resistance to apoptosis. Also, MMP-3 together with other MMPs are involved in both IGF-1-induced and TGF-induced bone defect healing, highlighting the involvement of MMPs in the process of bone formation and endochondral ossification [202,203].

MMP-3 is also involved in the regulation of the **receptor tyrosine kinase (RTK) pathway**, particularly through its effects on ECM components that bind RTK ligands [204,205]. For example, MMP-3 can cleave tenascin-C, an ECM glycoprotein that modulates cell adhesion and migration, releasing EGF-like repeats that activate RTKs. This cleavage event activates downstream signaling cascades, including the MAPK/ERK pathway, which promotes cell proliferation and survival [206]. The ability of MMP-3 to release RTK ligands further highlights its role as a key modulator of signaling pathways that govern cell behavior in both normal and pathological conditions.

Another pathway influenced by MMP-3 is the **renin-angiotensin system (RAS)**, which regulates blood pressure and fluid balance. MMP-3 has been implicated in the regulation of angiotensin II-induced myocardial fibrosis by modulating the activity of ECM components involved in collagen degradation [207]. By influencing the balance between ECM synthesis and degradation, MMP-3 affects the progression of myocardial fibrosis, highlighting its role in cardiovascular diseases.

The profound effects of MMP-3 indirectly regulation as a multifunctional enzyme exhibit as **activation of collagenases** to increase collagenolysis [208]. Collagenase MMP-1 can be activated after MMP-3 catalytic cleavage induced by tumor necrosis factor-α (TNF-α)-initiated collagenolysis resulting in pathological catabolism of skin collagen [17, 209].

### 4.4. MMP-3 in transcriptional regulation

In addition to its role as an extracellular enzyme, MMP-3 has been shown to participate in transcriptional regulation through intracellular signaling, adding a significant layer to its biological importance. MMP-3 influences various cellular processes such as stem cell differentiation, inflammation, tissue remodeling, and cancer progression [210]. There are two primary ways MMP-3 can translocate into the nucleus to exhibit transcriptional regulation: one involves immature MMP-3 remaining in the cytoplasm, and the other involves mature MMP-3 reentering the cell (Fig. 2).

For instance, MMP-3 can translocate into the nucleus due to a secretion signal sequence containing a helix-breaking proline at the fifth residue from the N-terminus. This proline weakens the signal peptide, similar to calreticulin, allowing a fraction of MMP-3 to remain inside the cell [211]. Other evidence suggests that MMP-3 translocate to the nucleus, where it exerts direct effects on gene transcription by identifying nuclear localization signals (NLS) in MMP-3, supporting its potential for nuclear functions [212]. Additionally, mature MMP-3 can be recycled back into cells via various endocytosis mechanisms, including α2β1 integrins, tetraspanin CD151, heparan sulfate proteoglycan CD44, cholesterol sulfate in lipid rafts, and low-density lipoprotein-related protein 1 [92,213,214]. Regardless of the mechanism by which MMP-3 reaches the nucleus, it has been shown to regulate the transcription of connective tissue growth factor (CTGF), a key regulator of fibrosis and ECM production, by binding to its promoter region [212]. MMP-3 also influences gene expression by interacting with key transcription factors such as NF-κB via its HPX domain, thereby altering the transcriptional landscape [215]. Furthermore, MMP-3 modulates the availability of signaling molecules that affect transcription factor activity. For example, by cleaving ECM components, MMP-3 releases growth factors such as TGF-β and fibroblast growth factor (FGF), which activate SMADs and other transcription factors involved in inflammation, tissue repair, and fibrosis [216,217].

The dual role of MMP-3 in both proteolytic and transcriptional regulation highlights its versatility as a mediator of cellular signaling. By interacting with various signaling pathways and transcriptional networks, MMP-3 plays a central role in coordinating cellular responses to environmental cues, making it a key player in both normal tissue homeostasis and disease progression.

MMP-3 exerts extensive influence across multiple molecular signaling networks by modulating extracellular matrix dynamics and activating key mediators such as TGF-β, RTKs, and Wnt/β-catenin signaling. Through direct cleavage of ECM components and liberation of membrane-bound growth factors, MMP-3 initiates cascades that drive processes like EMT, inflammation, and tumor progression. It also interacts with intracellular transcription factors, including NF-κB and AP-1, and participates in nuclear regulation of gene expression. The dual role of TGF-β in both repressing and activating MMP-3 highlights the context-dependent nature of its regulation. In various disease models, including fibrosis, cancer, and cardiovascular pathology, these MMP-3–mediated pathways converge to promote cellular plasticity, immune evasion, and tissue remodeling. Despite increasing mechanistic insights, most evidence remains disease- or cell-specific, with limited cross-validation. Understanding how MMP-3 selectively engages or bypasses these pathways in different tissues will be crucial for developing precise interventions. Overall, MMP-3 functions as a molecular hub linking extracellular proteolysis with intracellular signaling, reinforcing its relevance as a target for both diagnostic and therapeutic strategies.

## 5. Further perspectives and limitations

MMP-3 stands at a critical crossroads where molecular pathways converge to influence both physiological and pathological processes. This review has highlighted the broad spectrum of MMP-3’s molecular activities, underscoring how the enzyme’s ability to engage in both ECM-dependent and ECM-independent pathways links it to disease mechanisms. As the understanding of matrix metalloproteinase-3 (MMP-3) deepens, its role in infectious diseases is becoming increasingly evident, offering new avenues for research and therapeutic intervention. MMP-3’s involvement in viral infections, including its potential role in the tissue and vascular damage seen in severe cases of COVID-19, underscores its importance in immune response regulation. The enzyme’s ability to degrade ECM components and modulate inflammatory processes makes it a key player in viral pathogenesis, where excessive inflammation can lead to severe complications. Future research should focus on understanding how MMP-3 interacts with viral components and inflammatory mediators to drive tissue damage, with the goal of developing MMP-3 inhibitors to mitigate the harmful effects of hyper-inflammatory responses in viral infections. In addition, MMP-3’s role in bacterial infections, such as LPS-induced inflammation, is of particular interest. Studies have shown that MMP-3 is involved in the degradation of ECM components in response to bacterial endotoxins like LPS, contributing to tissue inflammation and immune dysregulation. Targeting MMP-3 in bacterial infections could be a promising strategy to reduce tissue damage and improve outcomes in conditions such as sepsis, where excessive matrix degradation exacerbates organ failure. The interplay between MMP-3 and other proteases, such as MMP-9, and its interactions with inflammatory mediators like TNF-α, suggest that targeting MMP-3 in combination with other molecules could provide a more comprehensive approach to managing infection-induced inflammation.

Beyond infectious diseases, MMP-3 genetic polymorphisms have emerged as important factors in determining susceptibility to a variety of conditions, including coronary artery disease (CAD) and inflammatory diseases. The continued exploration of MMP-3 genetic variants, particularly in diverse populations, could pave the way for personalized therapies that leverage genetic screening to predict disease risk and tailor treatments accordingly. Furthermore, biomarker research on MMP-3 is advancing, with the enzyme being explored as a reliable indicator for monitoring disease progression in various conditions, including viral and bacterial infections, as well as chronic inflammatory diseases like rheumatoid arthritis and cardiovascular disease. The use of MMP-3 as a biomarker in infectious diseases holds significant potential for guiding treatment strategies and assessing disease severity.

Despite these promising developments, several limitations remain in the study of MMP-3. One of the main challenges is the lack of specificity when targeting MMP-3 for therapeutic purposes. Given the structural similarity between MMP-3 and other matrix metalloproteinases, designing selective inhibitors remains difficult, raising the risk of off-target effects that may influence broader immune and tissue responses. This is particularly critical in infections, where balancing MMP-3 activity is necessary to avoid exacerbating tissue damage while supporting necessary immune functions. In infections, while MMP-3 has been identified as a contributor to tissue damage, especially in severe COVID-19 cases, its exact role in viral pathogenesis is still not fully understood. Research is needed to clarify whether targeting MMP-3 could effectively reduce tissue damage without impairing the body’s ability to fight the virus. Similarly, in bacterial infections, the use of MMP-3 as a therapeutic target is complicated by the variability in LPS-induced inflammation across different patient populations. Variations in circulating MMP-3 levels, influenced by factors such as disease severity and individual immune responses, make it challenging to standardize its use as a biomarker for bacterial infection severity or treatment efficacy. Another limitation is the conflicting results found in genetic studies on MMP-3 polymorphisms, particularly in relation to conditions such as CAD. While some studies have shown a strong association between specific polymorphisms and disease risk, others have reported no significant correlation, underscoring the need for larger, more diverse studies that can account for population-specific genetic variability.

Finally, translating preclinical findings from animal models to human settings continues to be a challenge. Although animal studies have provided valuable insights into MMP-3’s role in both infection and inflammation, the differences in immune regulation and MMP-3 activity between species must be addressed to develop effective human therapies. Further research is needed to bridge the gap between basic science and clinical application, especially in the context of infectious diseases. Moving ahead, MMP-3 research should prioritize unraveling the detailed mechanisms by which it mediates its effects across different diseases, with a focus on identifying more precise diagnostic tools and targeted therapies. At this molecular crossroads, MMP-3 holds significant potential for translating biological insights into clinical interventions, offering new opportunities for more effective diagnosis and treatment strategies in a variety of diseases.

## Figures and Tables

**Table 1:** MMP–3 applications in disease treatment and diagnosis.

Disease		MMP–3 Role	Diagnostic Potential	Therapeutic Application	Ref.
**Inflamma-tory Diseases**	Rheumatoid Arthritis (RA)	Contributes to joint inflammation and cartilage degradation	Serum MMP–3 as a biomarker for disease activity	*Erosive RA*: Monitoring MMP–3 levels to evaluate response to anti-TNF therapy	[65,66,72,74,75]
				Non-Erosive RA: Targeting MMP–3 to limit cartilage degradation	[72,82]
				*Early RA*: Monitoring MMP–3 levels for early diagnosis and treatment efficacy	[64,68,81,89]
				*Established RA:* MMP–3 levels used to assess long-term therapeutic responses	[63,71,79,80,82,85]
				*Methotrexate-Treated RA:* Monitoring MMP–3 to evaluate methotrexate response	[77,86]
				*Biologic-Treated RA:* MMP–3 levels to guide biologic therapy decisions	[71,77,78,84,85]
				*RA with Periodontitis:* Targeting MMP–3 to manage inflammation in both joints and gums	[70]
				*Juvenile Idiopathic Arthritis (JIA):* MMP–3 inhibition to prevent joint destruction in JIA	[73,91]
	Osteoarthritis (OA)	Degrades cartilage matrix components	MMP–3 levels correlate with joint degeneration	MMP–3 inhibition to slow OA progression	[65,86,87–90]
	Psoriatic Arthritis (PsA)	Mediates joint inflammation and damage	Elevated MMP–3 associated with joint damage	Potential use of MMP–3 as a biomarker for PsA	[91–94]
	Obesity	Influences adipose tissue inflammation	MMP–3 as a marker for chronic low-grade inflammation	Targeting MMP–3 to reduce metabolic dysfunction	[95–98,100]
	Pulmonary Inflammation	Drives ECM degradation and inflammation in lungs	MMP–3 as a biomarker for lung disease severity	Inhibition of MMP–3 to prevent tissue damage	[104,105,118]
	Dental Inflammation	Involved in inflammation of gingival tissues	MMP–3 as a biomarker for periodontitis	Targeting MMP–3 to reduce gum inflammation	[102]
	Systemic Lupus Erythematosus (SLE)	Contributes to tissue degradation and inflammation	MMP–3 as a marker for disease activity	Modulating MMP–3 to reduce systemic inflammation	[106–108]
**Infectious Disease**	COVID-19	Exacerbates inflammatory responses and tissue damage	MMP–3 as a biomarker for disease severity and progression	MMP–3 inhibition as a potential target to reduce lung damage and inflammation	[109–115]
	Bacterial Infections (e.g., Sepsis, Gram-negative Bacteria)	Contributes to ECM degradation and immune dysregulation	MMP–3 as a marker for systemic inflammation and sepsis severity	MMP–3 inhibition to modulate the immune response and prevent ECM degradation	[105,118,119]
	Fungal Infections (e.g., Cryptococcus)	Modulates chemokine expression and inflammation	MMP–3 as a marker for immune response and fungal invasion	MMP–3 inhibition to reduce pulmonary inflammation in fungal infections	[117,118]
	Viral Infections (Other than COVID-19)	Drives immune dysregulation and tissue remodeling	MMP–3 as a biomarker for tissue damage in viral infections	MMP–3 inhibition to reduce viral-induced tissue damage	[110,118]
	Parasitic Infections (e.g., Malaria)	Contributes to tissue destruction and immune dysregulation	MMP–3 as a marker for tissue damage in parasitic infections	Targeting MMP–3 to reduce ECM damage and inflammation	[118,119]
**Cardiovascular Diseases**	Coronary Artery Disease (CAD)	Genetic polymorphisms associated with CAD susceptibility	MMP–3 polymorphisms as potential genetic biomarkers for CAD	Understanding genetic influences could improve risk stratification and treatment	[120–124]
	Ischemic Heart Disease	MMP–3 levels linked to myocardial infarction and mortality	MMP–3 as a prognostic marker for cardiovascular outcomes	Monitoring MMP–3 levels to predict outcomes in ischemic heart disease patients	[125,126]
	Athero-sclerosis	Contributes to plaque instability and vascular remodeling	MMP–3 as a marker for atherosclerotic plaque vulnerability	Targeting MMP–3 to stabilize plaques and prevent adverse cardiovascular events	[127,128]
	Heart Failure	MMP–3 involved in myocardial remodeling	MMP–3 as a biomarker for heart failure severity	Therapeutic strategies to inhibit MMP–3 in heart failure management	[131]
	Peripheral Artery Disease (PAD)	MMP–3 implicated in endothelial dysfunction	MMP–3 as a marker for disease progression in PAD	Targeting MMP–3 to improve vascular health and function	[129,130]
	Stroke (Cerebro-vascular Accident)	Involved in post-stroke inflammation and neuronal damage	MMP–3 as a biomarker for stroke severity and recovery	Investigating MMP–3 as a therapeutic target in post-stroke treatment	[131]
**Neurodegenerative Diseases**	Alzheimer’s Disease	Contributes to neuroinflammation and blood-brain barrier (BBB) disruption	Biomarker for disease progression and BBB dysfunction	Target for therapies to prevent ECM degradation and neuroinflammation	[133,134,136–145]
	Parkinson’s Disease	Facilitates α-synuclein cleavage and dopaminergic cell death	Potential biomarker for non-motor symptoms	Targeted therapies to inhibit MMP–3 and prevent neurodegeneration	[146,147]
	Multiple Sclerosis	Modulates B-cell responses and ECM remodeling in neuroinflammatory lesions	Potential marker for disease activity and lesion development	MMP–3 inhibition to prevent ECM degradation and immune cell infiltration	[101,134,135]
	Ischemic Stroke	Contributes to delayed inflammation and neuronal death	Marker for post-stroke inflammation and brain damage	Target for neuroprotective therapies to reduce ECM degradation and inflammation	[101,103]
**Cancer**	Breast Cancer	Facilitates tumor invasion and metastasis	Biomarker for disease progression and recurrence	MMP–3 inhibition as a therapeutic target to control metastasis	[152–161]
	Prostate Cancer	Promotes tumor growth and angiogenesis	Marker for aggressive tumor behavior	Potential target to inhibit MMP–3-driven invasion and metastasis	[164]
	Bladder Cancer	Enhances invasive potential of cancer cells	Urinary MMP–3 as a biomarker for diagnosis	MMP–3 inhibition to limit tumor invasion and progression	[165–168]
	Lung Cancer	Promotes tumor growth and metastasis	Prognostic marker for poor outcomes	Targeted therapies to inhibit MMP–3 and suppress metastasis	[169]
	Oral and Oropharyngeal Cancer	Contributes to ECM degradation and cancer invasion	Biomarker for tumor aggressiveness	MMP–3 inhibition as a potential treatment for limiting tumor growth	[162,163]
	Colon Cancer	Enhances ECM breakdown, aiding in metastasis	Biomarker for metastatic potential	MMP–3 as a therapeutic target to prevent invasion	[155]
	Ovarian Cancer	Drives tumor progression	Marker for disease progression	MMP–3 inhibition to prevent tumor growth and metastasis	[170]

## Data Availability

No data was used for the research described in the article.

## References

[1] Honsawek S, , Association of MMP-3 (−1612 5A/6A) polymorphism with knee osteoarthritis in Thai population, Rheuma Int 33 (2) (2013) 435–439. 10.1007/s00296-012-2371-y 22457004

[2] Jabłońska-Trypuć A, Matejczyk M, Rosochacki S, Matrix metalloproteinases (MMPs), the main extracellular matrix (ECM) enzymes in collagen degradation, as a target for anticancer drugs, J. Enzym. Inhib. Med Chem 31 (sup1) (2016) 177–183. 10.3109/14756366.2016.1161620 27028474

[3] Molière S, , Roles of matrix metalloproteinases and their natural inhibitors in metabolism: insights into health and disease, Int J. Mol. Sci 24 (13) (2023). 10.3390/ijms241310649 PMC10341707 37445827

[4] Kreis T, Vale R, Guidebook to the extracellular matrix, anchor, and adhesion proteins, Sambrook and Tooze Publications, 1999.

[5] Sugiyama E, [Role of matrix metalloproteinase-3 in joint destruction in rheumatoid arthritis], Clin. Calcium 17 (4) (2007) 528–534. 17404481

[6] Yamanaka H, Usefulness of serum matrix metalloproteinase-3(MMP-3) level in the diagnosis of rheumatoid arthritis, Nihon Rinsho 60 (12) (2002) 2325–2330. 12510357

[7] Holliday DL, , Intrinsic genetic characteristics determine tumor-modifying capacity of fibroblasts: matrix metalloproteinase-3 5A/5A genotype enhances breast cancer cell invasion, Breast Cancer Res 9 (5) (2007) R67. 17922906 10.1186/bcr1775 PMC2242664

[8] Hinoda Y, , Association of functional polymorphisms of matrix metalloproteinase (MMP)-1 and MMP-3 genes with colorectal cancer, Int J. Cancer 102 (5) (2002) 526–529. 12432557 10.1002/ijc.10750

[9] Mehner C, , Tumor cell expression of MMP3 as a prognostic factor for poor survival in pancreatic, pulmonary, and mammary carcinoma, Genes Cancer 6 (11–12) (2015) 480–489. 26807201 10.18632/genesandcancer.90 PMC4701227

[10] Flores-Pliego A, , Matrix metalloproteinase-3 (MMP-3) is an endogenous activator of the MMP-9 secreted by placental leukocytes: implication in human labor, PLoS One 10 (12) (2015) e0145366. 26713439 10.1371/journal.pone.0145366 PMC4699849

[11] Khokha R, Murthy A, Weiss A, Metalloproteinases and their natural inhibitors in inflammation and immunity, Nat. Rev. Immunol 13 (9) (2013) 649–665. 23969736 10.1038/nri3499

[12] Stamenkovic I, Extracellular matrix remodelling: the role of matrix metalloproteinases, J. Pathol 200 (4) (2003) 448–464. 12845612 10.1002/path.1400

[13] Vandenbroucke RE, Libert C, Is there new hope for therapeutic matrix metalloproteinase inhibition? Nat. Rev. Drug Discov 13 (12) (2014) 904–927. 25376097 10.1038/nrd4390

[14] Shi Y, , Matrix metalloproteinase inhibitors (MMPIs) as attractive therapeutic targets: recent progress and current challenges, NanoImpact 21 (2021) 100293. 35559782 10.1016/j.impact.2021.100293

[15] Almutairi S, , Matrix metalloproteinases inhibitors in cancer treatment: an updated review (2013–2023), Molecules 28 (14) (2023). 10.3390/molecules28145567 PMC10384300 37513440

[16] Madzharova E, , Post-translational modification-dependent activity of matrix metalloproteinases, Int J. Mol. Sci 20 (12) (2019). 10.3390/ijms20123077 PMC6627178 31238509

[17] Mirastschijski U, , Matrix metalloproteinase-3 is key effector of TNF-α-induced collagen degradation in skin, Int J. Mol. Sci 20 (20) (2019). 10.3390/ijms20205234 PMC6829232 31652545

[18] Petrella BL, Armstrong DA, Vincenti MP, Interleukin-1 beta and transforming growth factor-beta 3 cooperate to activate matrix metalloproteinase expression and invasiveness in A549 lung adenocarcinoma cells, Cancer Lett. 325 (2) (2012) 220–226. 22796605 10.1016/j.canlet.2012.07.009

[19] Peeters-Joris C, Hammani K, Singer CF, Differential regulation of MMP-13 (collagenase-3) and MMP-3 (stromelysin-1) in mouse calvariae, Biochim Biophys Acta 1405 (1–3) (1998) 14–28. 9784593 10.1016/s0167-4889(98)00094-9

[20] Giraudon P, , Cytokines secreted by glial cells infected with HTLV-I modulate the expression of matrix metalloproteinases (MMPs) and their natural inhibitor (TIMPs): possible involvement in neurodegenerative processes, Mol. Psychiatry 2 (2) (1997) 107–110, 84. 9106228 10.1038/sj.mp.4000218

[21] Borghaei RC, Sullivan C, Mochan E, Identification of a cytokine-induced repressor of interleukin-1 stimulated expression of stromelysin 1 (MMP-3), J. Biol. Chem 274 (4) (1999) 2126–2131. 9890974 10.1074/jbc.274.4.2126 PMC1595537

[22] Wang X, , Tumor necrosis factor-α- and interleukin-1β-dependent matrix metalloproteinase-3 expression in nucleus pulposus cells requires cooperative signaling via syndecan 4 and mitogen-activated protein kinase-NF-κB axis: implications in inflammatory disc disease, Am. J. Pathol 184 (9) (2014) 2560–2572. 25063530 10.1016/j.ajpath.2014.06.006 PMC4188173

[23] Fleenor DL, Pang IH, Clark AF, Involvement of AP-1 in interleukin-1alpha-stimulated MMP-3 expression in human trabecular meshwork cells, Invest Ophthalmol. Vis. Sci 44 (8) (2003) 3494–3501. 12882799 10.1167/iovs.02-0757

[24] He Z, , Interleukin 1 beta and matrix metallopeptidase 3 contribute to development of epidermal growth factor receptor-dependent serrated polyps in mouse cecum, Gastroenterology 157 (6) (2019) 1572–1583.e8. 31470007 10.1053/j.gastro.2019.08.025 PMC7006742

[25] Kim JM, , Reversine inhibits MMP-1 and MMP-3 expressions by suppressing of ROS/MAPK/AP-1 activation in UV-stimulated human keratinocytes and dermal fibroblasts, Exp. Dermatol 27 (3) (2018) 298–301. 29341262 10.1111/exd.13494

[26] Song HK, , Reversine inhibits MMP-3, IL-6 and IL-8 expression through suppression of ROS and JNK/AP-1 activation in interleukin-1β-stimulated human gingival fibroblasts, Arch. Oral. Biol 108 (2019) 104530. 31470141 10.1016/j.archoralbio.2019.104530

[27] Sekine C, Nanki T, Yagita H, Macrophage-derived delta-like protein 1 enhances interleukin-6 and matrix metalloproteinase 3 production by fibroblast-like synoviocytes in mice with collagen-induced arthritis, Arthritis Rheuma 66 (10) (2014) 2751–2761. 10.1002/art.38743 24943093

[28] Cutler SJ, , Novel STAT binding elements mediate IL-6 regulation of MMP-1 and MMP-3, Sci. Rep 7 (1) (2017) 8526. 28819304 10.1038/s41598-017-08581-y PMC5561029

[29] Ganguly SS, , Notch3 promotes prostate cancer-induced bone lesion development via MMP-3, Oncogene 39 (1) (2020) 204–218. 31467432 10.1038/s41388-019-0977-1 PMC6938550

[30] Qin Y, , Age-associated B cells contribute to the pathogenesis of rheumatoid arthritis by inducing activation of fibroblast-like synoviocytes via TNF-α-mediated ERK1/2 and JAK-STAT1 pathways, Ann. Rheum. Dis 81 (11) (2022) 1504–1514. 35760450 10.1136/ard-2022-222605

[31] Seki I, , Concomitant treatment with etanercept and tacrolimus synergistically attenuates arthritis progression via inhibition of matrix metalloproteinase-3 production and osteoclastogenesis in human TNF-α transgenic mice, Mediat. Inflamm 2019 (2019) 4176974. 10.1155/2019/4176974 PMC6942915 31949424

[32] Sun P, Mu Y, Zhang S, A novel NF-κB/MMP-3 signal pathway involves in the aggressivity of glioma promoted by Bmi-1, Tumour Biol. 35 (12) (2014) 12721–12727. 25252846 10.1007/s13277-014-2597-2

[33] Tang CH, , Involvement of matrix metalloproteinase-3 in CCL5/CCR5 pathway of chondrosarcomas metastasis, Biochem Pharm. 79 (2) (2010) 209–217. 19682436 10.1016/j.bcp.2009.08.006

[34] Hou SM, Hou CH, Liu JF, CX3CL1 promotes MMP-3 production via the CX3CR1, c-Raf, MEK, ERK, and NF-κB signaling pathway in osteoarthritis synovial fibroblasts, Arthritis Res Ther. 19 (1) (2017) 282. 29268768 10.1186/s13075-017-1487-6 PMC5740560

[35] Banik P, , A computational study to assess the polymorphic landscape of matrix metalloproteinase 3 promoter and its effects on transcriptional activity, Comput. Biol. Med 145 (2022) 105404. 35339097 10.1016/j.compbiomed.2022.105404

[36] Hoberg M, , Attachment to laminin-111 facilitates transforming growth factor beta-induced expression of matrix metalloproteinase-3 in synovial fibroblasts, Ann. Rheum. Dis 66 (4) (2007) 446–451. 17124250 10.1136/ard.2006.060228 PMC1856036

[37] Di Sabatino A, , Blockade of transforming growth factor beta upregulates T-box transcription factor T-bet, and increases T helper cell type 1 cytokine and matrix metalloproteinase-3 production in the human gut mucosa, Gut 57 (5) (2008) 605–612. 18178611 10.1136/gut.2007.130922

[38] Yang H, , TGF-β1 antagonizes TNF-α induced up-regulation of matrix metalloproteinase 3 in nucleus pulposus cells: role of the ERK1/2 pathway, Connect Tissue Res 56 (6) (2015) 461–468. 26075533 10.3109/03008207.2015.1054030

[39] Borghaei RC, , NF-kappaB binds to a polymorphic repressor element in the MMP-3 promoter, Biochem Biophys. Res Commun 316 (1) (2004) 182–188. 15003528 10.1016/j.bbrc.2004.02.030

[40] Borghaei RC, Gorski G, Javadi M, NF-kappaB and ZBP-89 regulate MMP-3 expression via a polymorphic site in the promoter, Biochem Biophys. Res Commun 382 (2) (2009) 269–273. 19275880 10.1016/j.bbrc.2009.03.002 PMC2678966

[41] Hsieh CL, , Reactive oxygen species-mediated switching expression of MMP-3 in stromal fibroblasts and cancer cells during prostate cancer progression, Sci. Rep 7 (1) (2017) 9065. 28831065 10.1038/s41598-017-08835-9 PMC5567216

[42] Sawamura D, , Involvement of the AP-1 site within the 5′-flanking region of the stromelysin-1 gene in induction of the gene expression by UVA irradiation, Arch. Dermatol. Res 288 (10) (1996) 628–632. 8919048 10.1007/BF02505268

[43] Pang IH, , Aqueous outflow-enhancing effect of tert-butylhydroquinone: involvement of AP-1 activation and MMP-3 expression, Invest Ophthalmol. Vis. Sci 44 (8) (2003) 3502–3510. 12882800 10.1167/iovs.02-0758

[44] White LA, Maute C, Brinckerhoff CE, ETS sites in the promoters of the matrix metalloproteinases collagenase (MMP-1) and stromelysin (MMP-3) are auxiliary elements that regulate basal and phorbol-induced transcription, Connect Tissue Res 36 (4) (1997) 321–335. 9610890 10.3109/03008209709160231

[45] Baillat D, , ETS-1 transcription factor binds cooperatively to the palindromic head to head ETS-binding sites of the stromelysin-1 promoter by counteracting autoinhibition, J. Biol. Chem 277 (33) (2002) 29386–29398. 12034715 10.1074/jbc.M200088200

[46] Baillat D, , Stromelysin-1 expression is activated in vivo by Ets-1 through palindromic head-to-head Ets binding sites present in the promoter, Oncogene 25 (42) (2006) 5764–5776. 16652151 10.1038/sj.onc.1209583

[47] Leprivier G, , Ets-1 p51 and p42 isoforms differentially modulate Stromelysin-1 promoter according to induced DNA bend orientation, Nucleic Acids Res 37 (13) (2009) 4341–4352. 19465391 10.1093/nar/gkp307 PMC2715226

[48] Horváth B, , Expression of ets-1 transcription factor in human head and neck squamous cell carcinoma and effect of histamine on metastatic potential of invasive tumor through the regulation of expression of ets-1 and matrix metalloproteinase-3, Head. Neck 27 (7) (2005) 585–596. 15887216 10.1002/hed.20188

[49] Bartel DP, MicroRNAs: target recognition and regulatory functions, Cell 136 (2) (2009) 215–233. 19167326 10.1016/j.cell.2009.01.002 PMC3794896

[50] Jia D, , miR-146a-5p expression is upregulated by the CXCR4 antagonist TN14003 and attenuates SDF-1-induced cartilage degradation, Mol. Med Rep 19 (5) (2019) 4388–4400. 30942441 10.3892/mmr.2019.10076 PMC6472139

[51] Korotkov A, , Increased expression of matrix metalloproteinase 3 can be attenuated by inhibition of microRNA-155 in cultured human astrocytes, J. Neuroinflamm 15 (1) (2018) 211. 10.1186/s12974-018-1245-y PMC6054845 30031401

[52] Quiñones-Díaz BI, , MicroRNA-18a-5p suppresses tumor growth via targeting matrix metalloproteinase-3 in cisplatin-resistant ovarian cancer, Front Oncol. 10 (2020) 602670. 33392094 10.3389/fonc.2020.602670 PMC7774672

[53] Deng L, , MicroRNA-613 enhances nasopharyngeal carcinoma cell radiosensitivity via the DNA methyltransferase 3B/tissue inhibitor of matrix metalloproteinase-3/signal transducer and activator of transcription-1/forkhead box O-1 axis, Dis. Markers 2022 (2022) 5699275. 36061358 10.1155/2022/5699275 PMC9439912

[54] Nagase H, , Stepwise activation mechanisms of the precursor of matrix metalloproteinase 3 (stromelysin) by proteinases and (4-aminophenyl)mercuric acetate, Biochemistry 29 (24) (1990) 5783–5789. 2383557 10.1021/bi00476a020

[55] Ra HJ, Parks WC, Control of matrix metalloproteinase catalytic activity, Matrix Biol. 26 (8) (2007) 587–596. 17669641 10.1016/j.matbio.2007.07.001 PMC2246078

[56] Sternlicht MD, Werb Z, How matrix metalloproteinases regulate cell behavior, Annu Rev. Cell Dev. Biol 17 (2001) 463–516. 11687497 10.1146/annurev.cellbio.17.1.463 PMC2792593

[57] Thibeaux R, , The parasite Entamoeba histolytica exploits the activities of human matrix metalloproteinases to invade colonic tissue, Nat. Commun 5 (2014) 5142. 25291063 10.1038/ncomms6142

[58] Gomez DE, , Tissue inhibitors of metalloproteinases: structure, regulation and biological functions, Eur. J. Cell Biol 74 (2) (1997) 111–122. 9352216

[59] Kontogiorgis CA, Papaioannou P, Hadjipavlou-Litina DJ, Matrix metalloproteinase inhibitors: a review on pharmacophore mapping and (Q)SARs results, Curr. Med Chem 12 (3) (2005) 339–355. 15723623 10.2174/0929867053363243

[60] Shi L, , Matrix Metalloproteinase-3 induces proteoglycan degradation in gouty arthritis model, Gene 765 (2021) 145120. 32896590 10.1016/j.gene.2020.145120

[61] Wei JC, , Genetic polymorphisms of the matrix metalloproteinase-3 (MMP-3) and tissue inhibitors of matrix metalloproteinases-1 (TIMP-1) modulate the development of ankylosing spondylitis, Ann. Rheum. Dis 68 (11) (2009) 1781–1786. 19019896 10.1136/ard.2008.099481

[62] Tabandeh MR, Oryan A, Mohammadalipour A, Polysaccharides of aloe vera induce MMP-3 and TIMP-2 gene expression during the skin wound repair of rat, Int J. Biol. Macromol 65 (2014) 424–430. 24491493 10.1016/j.ijbiomac.2014.01.055

[63] Sun S, , The active form of MMP-3 is a marker of synovial inflammation and cartilage turnover in inflammatory joint diseases, BMC Musculoskelet. Disord 15 (2014) 93. 24641725 10.1186/1471-2474-15-93 PMC4003863

[64] Shinozaki M, , Elevation of serum matrix metalloproteinase-3 as a predictive marker for the long-term disability of rheumatoid arthritis patients in a prospective observational cohort IORRA, Mod. Rheuma 17 (5) (2007) 403–408. 10.1007/s10165-007-0608-5 17929133

[65] Kobayashi A, , Serum levels of matrix metalloproteinase 3 (stromelysin 1) for monitoring synovitis in rheumatoid arthritis, Arch. Pathol. Lab Med 131 (4) (2007) 563–570. 17425385 10.5858/2007-131-563-SLOMMS

[66] Tsushima H, , CCAAT/enhancer binding protein β regulates expression of matrix metalloproteinase-3 in arthritis, Ann. Rheum. Dis 71 (1) (2012) 99–107. 21917825 10.1136/annrheumdis-2011-200061

[67] Gorai M, , Weighting with the Lansbury articular index improves the correlation of ultrasound score with serum matrix metalloproteinase-3 level in rheumatoid arthritis patients, Mod. Rheuma 24 (6) (2014) 915–919. 10.3109/14397595.2014.888794 24670135

[68] Takeshita M, , Alteration of matrix metalloproteinase-3 O-glycan structure as a biomarker for disease activity of rheumatoid arthritis, Arthritis Res Ther. 18 (1) (2016) 112. 27209430 10.1186/s13075-016-1013-2 PMC4875599

[69] Zhou L, , Matrix metalloproteinase-3 and the 7-joint ultrasound score in the assessment of disease activity and therapeutic efficacy in patients with moderate to severe rheumatoid arthritis, Arthritis Res Ther. 19 (1) (2017) 250. 29141665 10.1186/s13075-017-1449-z PMC5688630

[70] Zhao Y, , [Expression of matrix metalloproteinase-3 in patients with reheumatoid arthritis and its correlation with chronic periodontitis and rheumatoid arthritis], Zhonghua Kou Qiang Yi Xue Za Zhi 54 (3) (2019) 164–169. 30856693 10.3760/cma.j.issn.1002-0098.2019.03.004

[71] Brennan FM, , Reduction of serum matrix metalloproteinase 1 and matrix metalloproteinase 3 in rheumatoid arthritis patients following anti-tumour necrosis factor-alpha (cA2) therapy, Br. J. Rheuma 36 (6) (1997) 643–650. 10.1093/rheumatology/36.6.643 9236673

[72] Shovman O, , The diagnostic utility of anti-cyclic citrullinated peptide antibodies, matrix metalloproteinase-3, rheumatoid factor, erythrocyte sedimentation rate, and C-reactive protein in patients with erosive and non-erosive rheumatoid arthritis, Clin. Dev. Immunol 12 (3) (2005) 197–202. 16295525 10.1080/17402520500233510 PMC2275423

[73] Kamphuis S, , Novel self-epitopes derived from aggrecan, fibrillin, and matrix metalloproteinase-3 drive distinct autoreactive T-cell responses in juvenile idiopathic arthritis and in health, Arthritis Res Ther. 8 (6) (2006) R178. 10.1186/ar2088 PMC1794523 17129378

[74] Nasution MES, Haryuna TSH, Elevated matrix metalloproteinase-3 level may affect hearing function in patients with rheumatoid arthritis, J. Chin. Med Assoc 82 (4) (2019) 272–276. 30893257 10.1097/JCMA.0000000000000036

[75] Tuncer T, , Matrix metalloproteinase-3 levels in relation to disease activity and radiological progression in rheumatoid arthritis, Adv. Clin. Exp. Med 28 (5) (2019) 665–670. 30740946 10.17219/acem/94065

[76] Liang Z, , Evaluation of the immune feature of ACPA-negative rheumatoid arthritis and the clinical value of matrix metalloproteinase-3, Front Immunol. 13 (2022) 939265. 35967336 10.3389/fimmu.2022.939265 PMC9363571

[77] Yeo J, , Evaluation of serum matrix metalloproteinase-3 as an objective indicator for the disease activity in rheumatoid arthritis patients treated with methotrexate versus tocilizumab: 24-week results from a prospective randomized controlled study, J. Rheum. Dis 29 (2) (2022) 89–97. 37475900 10.4078/jrd.2022.29.2.89 PMC10351360

[78] Hussein R, Aboukhamis I, Serum matrix metalloproteinase-3 levels monitor the therapeutic efficacy in Syrian patients with rheumatoid arthritis, Heliyon 9 (3) (2023) e14008. 36895354 10.1016/j.heliyon.2023.e14008 PMC9989645

[79] Tokai N, , Correction: Serum matrix metalloproteinase 3 levels are associated with an effect of iguratimod as add-on therapy to biological DMARDs in patients with rheumatoid arthritis, PLoS One 14 (1) (2019) e0211750. 30699195 10.1371/journal.pone.0211750 PMC6353179

[80] Mamehara A, , Serum matrix metalloproteinase-3 as predictor of joint destruction in rheumatoid arthritis, treated with non-biological disease modifying anti-rheumatic drugs, Kobe J. Med Sci 56 (3) (2010) E98–E107. 21063156

[81] Ma J, , Value of serum matrix metalloproteinase-3 in the assessment of active disease in patients with rheumatoid arthritis, Zhonghua Yi Xue Za Zhi 95 (47) (2015) 3823–3828. 27337798

[82] Hattori Y, Kida D, Kaneko A, Normal serum matrix metalloproteinase-3 levels can be used to predict clinical remission and normal physical function in patients with rheumatoid arthritis, Clin. Rheuma 38 (1) (2019) 181–187. 10.1007/s10067-017-3829-9 28940139

[83] Lee R, , Fluorogenic probe for detecting active matrix metalloproteinase-3 (MMP-3) in plasma and peripheral blood neutrophils to indicate the severity of rheumatoid arthritis, ACS Biomater. Sci. Eng 5 (6) (2019) 3039–3048. 33405657 10.1021/acsbiomaterials.9b00084

[84] Takemoto T, , Improvement in matrix metalloproteinase-3 independently predicts low disease activity at 52 weeks in bio-switch rheumatoid arthritis patients treated with abatacept, Clin. Exp. Rheuma 38 (5) (2020) 933–939. 32083543

[85] Tokai N, , Serum matrix metalloproteinase 3 levels are associated with an effect of iguratimod as add-on therapy to biological DMARDs in patients with rheumatoid arthritis, PLoS One 13 (8) (2018) e0202601. 30138480 10.1371/journal.pone.0202601 PMC6107217

[86] Ateş A, , Serum pro-matrix metalloproteinase-3 as an indicator of disease activity and severity in rheumatoid arthritis: comparison with traditional markers, Rheuma Int 27 (8) (2007) 715–722. 10.1007/s00296-007-0338-1 17426976

[87] Gotoh H, , Levels of matrix metalloproteinase-3 and urokinase-type plasminogen activator in knee synovial fluids from patients with rheumatoid arthritis and osteoarthritis, Ryumachi 37 (1) (1997) 3–8. 9128417

[88] Kamata Y, Minota S, No increase in synovial fluid level of matrix metalloproteinase-3 by systemic administration of glucocorticoids in rheumatoid arthritis, Eur. J. Intern Med 26 (5) (2015) 371–372. 25820020 10.1016/j.ejim.2015.03.006

[89] Liu MK, Wang LC, Hu FL, Value of serum matrix metalloproteinase 3 in the assessment of early rheumatoid arthritis, Beijing Da Xue Xue Bao Yi Xue Ban. 50 (6) (2018) 981–985. 30562768

[90] Zhao X, , Inhibitory effects of IL-6-mediated matrix metalloproteinase-3 and −13 by Achyranthes japonica nakai root in osteoarthritis and rheumatoid arthritis mice models, Pharmaceuticals 14 (8) (2021). 10.3390/ph14080776 PMC8402178 34451873

[91] Geneva-Popova M, , Assessment of serum and synovial fluid MMP-3 and MPO as biomarkers for psoriatic arthritis and their relation to disease activity indices, Rheuma Int 42 (9) (2022) 1605–1615. 10.1007/s00296-022-05159-4 35708757

[92] Lindqvist U, Phil-Lundin I, Engström-Laurent A, Dermal distribution of hyaluronan in psoriatic arthritis; coexistence of CD44, MMP3 and MMP9, Acta Derm. Venereol 92 (4) (2012) 372–377. 22278305 10.2340/00015555-1286

[93] Xia L, , Increased serum TWEAK levels in Psoriatic arthritis: relationship with disease activity and matrix metalloproteinase-3 serum levels, Cytokine 53 (3) (2011) 289–291. 21190865 10.1016/j.cyto.2010.12.003

[94] Fraser AR, , Lipoma arborescens co-existing with psoriatic arthritis releases tumour necrosis factor alpha and matrix metalloproteinase 3, Ann. Rheum. Dis 69 (4) (2010) 776–777. 20237127 10.1136/ard.2008.106047

[95] Wu Y, , High-fat diet-induced obesity regulates MMP3 to modulate depot- and sex-dependent adipose expansion in C57BL/6J mice, Am. J. Physiol. Endocrinol. Metab 312 (1) (2017) E58–E71. 27879248 10.1152/ajpendo.00128.2016 PMC5283879

[96] Boumiza S, , Role of MMP-1 (−519A/G, −1607 1G/2G), MMP-3 (Lys45Glu), MMP-7 (−181A/G), and MMP-12 (−82A/G) variants and plasma MMP levels on obesity-related phenotypes and microvascular reactivity in a Tunisian population, Dis. Markers 2017 (2017) 6198526. 29317790 10.1155/2017/6198526 PMC5727656

[97] Williams RC, , Leptin and pro-inflammatory stimuli synergistically upregulate MMP-1 and MMP-3 secretion in human gingival fibroblasts, PLoS One 11 (2) (2016) e0148024. 26829555 10.1371/journal.pone.0148024 PMC4734666

[98] Serretta V, , Clinical and biochemical markers of visceral adipose tissue activity: Body mass index, visceral adiposity index, leptin, adiponectin, and matrix metalloproteinase-3. Correlation with Gleason patterns 4 and 5 at prostate biopsy, Urol. Ann 10 (3) (2018) 280–286. 30089986 10.4103/UA.UA_188_17 PMC6060586

[99] Maquoi E, , Enhanced nutritionally induced adipose tissue development in mice with stromelysin-1 gene inactivation, Thromb. Haemost 89 (4) (2003) 696–704. 12669125

[100] Traurig MT, , Differential expression of matrix metalloproteinase 3 (MMP3) in preadipocytes/stromal vascular cells from nonobese nondiabetic versus obese nondiabetic Pima Indians, Diabetes 55 (11) (2006) 3160–3165. 17065356 10.2337/db06-0373

[101] Walker EJ, Rosenberg GA, TIMP-3 and MMP-3 contribute to delayed inflammation and hippocampal neuronal death following global ischemia, Exp. Neurol 216 (1) (2009) 122–131. 19111539 10.1016/j.expneurol.2008.11.022 PMC2709713

[102] Goda S, , Effects of JNK1/2 on the inflammation cytokine TNF-α-enhanced production of MMP-3 in human dental pulp fibroblast-like cells, Int Endod. J 48 (12) (2015) 1122–1128. 25393585 10.1111/iej.12411

[103] Van Hove I, , MMP-3 deficiency alleviates endotoxin-induced acute inflammation in the posterior eye segment, Int J. Mol. Sci 17 (11) (2016). 10.3390/ijms17111825 PMC5133826 27809288

[104] Zhang Y, , MMP-3 mediates copper oxide nanoparticle-induced pulmonary inflammation and fibrosis, J. Nanobiotechnol 22 (1) (2024) 428. 10.1186/s12951-024-02707-x PMC11264740 39030581

[105] Roy S, , Regulation of vascular responses to inflammation: inducible matrix metalloproteinase-3 expression in human microvascular endothelial cells is sensitive to antiinflammatory Boswellia, Antioxid. Redox Signal 8 (3–4) (2006) 653–660. 16677108 10.1089/ars.2006.8.653

[106] Kotajima L, , Increased levels of matrix metalloproteinase-3 in sera from patients with active lupus nephritis, Clin. Exp. Rheuma 16 (4) (1998) 409–415. 9706420

[107] Gheita TA, , Clinical significance of matrix metalloproteinase-3 in systemic lupus erythematosus patients: a potential biomarker for disease activity and damage, Acta Reum. Port 40 (2) (2015) 145–149. 26219967

[108] Lee JM, , Association between serum matrix metalloproteinase- (MMP-) 3 levels and systemic lupus erythematosus: a meta-analysis, Dis. Markers 2019 (2019) 9796735. 31396295 10.1155/2019/9796735 PMC6668546

[109] Toczyłowski KA-O, , Differential Inflammatory Responses in Adult and Pediatric COVID-19 Patients: Implications for Long-Term Consequences and Anti-Inflammatory Treatment. 2024(1643–3750 (Electronic)). 10.12659/MSM.944052 PMC11149468 38816982

[110] Ramezani S, , Association of the matrix metalloproteinases (MMPs) family gene polymorphisms and the risk of coronavirus disease 2019 (COVID-19); implications of contribution for development of neurological symptoms in the COVID-19 patients, Mol. Biol. Rep 50 (1) (2023) 173–183. 36319784 10.1007/s11033-022-07907-y PMC9628292

[111] Gelzo M, , Matrix metalloproteinases (MMP) 3 and 9 as biomarkers of severity in COVID-19 patients. 2022(2045–2322 (Electronic)). 10.1038/s41598-021-04677-8 PMC8786927 35075175

[112] Kadry R, Newsome AS, and Somanath PR, Pharmacological Inhibition of MMP3 as a Potential Therapeutic Option for COVID-19 Associated Acute Respiratory Distress Syndrome. 2021(2212–3989 (Electronic)). 10.2174/1871526520666201116100310 PMC8551813 33200717

[113] Almuntashiri S, , MMP3 in Severe COVID-19: A Biomarker or Therapeutic Target? 2023(2212–3989 (Electronic)). 10.2174/1871526522666220619121539 PMC11042506 35726419

[114] Shi S, , Matrix metalloproteinase 3 as a valuable marker for patients with COVID-19, J. Med Virol 93 (1) (2021) 528–532. 32603484 10.1002/jmv.26235 PMC7362036

[115] Lee HA-O and Kim WA-O, The Role of Matrix Metalloproteinase in Inflammation with a Focus on Infectious Diseases. LID - 10.3390/ijms231810546 [doi] LID - 10546. 2022 (1422–0067 (Electronic)). PMC9500641 36142454

[116] Tuharov Y, , Plasma levels of MMPs and TIMP-1 in patients with osteoarthritis after recovery from COVID-19, Rev. Recent Clin. Trials 18 (2) (2023) 123–128. 37231778 10.2174/1574887118666230131141608

[117] Supasorn O, , Matrix metalloproteinases contribute to the regulation of chemokine expression and pulmonary inflammation in Cryptococcus infection, Clin. Exp. Immunol 183 (3) (2016) 431–440. 26445891 10.1111/cei.12725 PMC4750603

[118] Vanlaere I, Libert C, Matrix metalloproteinases as drug targets in infections caused by gram-negative bacteria and in septic shock, Clin. Microbiol Rev 22 (2) (2009) 224–239 (Table of Contents). 19366913 10.1128/CMR.00047-08 PMC2668236

[119] Van den Steen PE, , Matrix metalloproteinases, tissue inhibitors of MMPs and TACE in experimental cerebral malaria, Lab Invest 86 (9) (2006) 873–888. 16865090 10.1038/labinvest.3700454

[120] Humphries SE, The stromelysin-1 (MMP-3) gene and risk of coronary artery disease: a candidate gene that has won the election, Thromb. Haemost 90 (1) (2003) 3–6. 12876619

[121] Ye S, Influence of matrix metalloproteinase genotype on cardiovascular disease susceptibility and outcome, Cardiovasc Res 69 (3) (2006) 636–645. 16122719 10.1016/j.cardiores.2005.07.015

[122] Wenwang L, Susceptibility of MMP3 gene polymorphism to coronary artery disease: a meta-analysis, J. Med Biochem 42 (4) (2023) 685–693. 38084252 10.5937/jomb0-43315 PMC10710786

[123] Shalia KK, , Matrix metalloproteinase-3 (MMP-3) −1612 5A/6A promoter polymorphism in coronary artery disease in Indian population, Indian J. Clin. Biochem 25 (2) (2010) 133–140. 23105899 10.1007/s12291-010-0025-y PMC3453096

[124] McGlinchey PG, , The matrix metalloproteinase-3 (MMP-3) 5A/6A promoter polymorphism is not associated with ischaemic heart disease: analysis employing a family based approach, Dis. Markers 20 (6) (2004) 289–294. 15665388 10.1155/2004/715745 PMC3839324

[125] Wu TC, , Plasma matrix metalloproteinase-3 level is an independent prognostic factor in stable coronary artery disease, Eur. J. Clin. Invest 35 (9) (2005) 537–545. 16128859 10.1111/j.1365-2362.2005.01548.x

[126] Guizani I, , Matrix metalloproteinase-3 predicts clinical cardiovascular outcomes in patients with coronary artery disease: a 5 years cohort study, Mol. Biol. Rep 46 (5) (2019) 4699–4707. 31218540 10.1007/s11033-019-04914-4

[127] Guizani I, , Matrix metalloproteinase 3 and 9 as genetic biomarkers for the occurrence of cardiovascular complications in coronary artery disease: a prospective cohort study, Mol. Biol. Rep 49 (10) (2022) 9171–9179. 35960412 10.1007/s11033-022-07742-1

[128] Krumsiek A, Kropf S, Gardemann A, Tumor necrosis factor-alpha G(−308)A promoter polymorphism, matrix metalloproteinase (MMP)-3 5A/6A gene variation, MMP-9C(−1562)T promoter polymorphism and risk and extent of ischemic heart disease, Clin. Chem. Lab Med 46 (2) (2008) 292–295. 18076359 10.1515/CCLM.2008.027

[129] Ghaffarzadeh A, , Association of MMP-1 (rs1799750)-1607 2G/2G and MMP-3 (rs3025058)-1612 6A/6A genotypes with coronary artery disease risk among iranian turks, J. Cardiovasc Pharm 74 (5) (2019) 420–425. 10.1097/FJC.0000000000000727 31356534

[130] Beton O, , Association between MMP-3 and MMP-9 polymorphisms and coronary artery disease, Biomed. Rep 5 (6) (2016) 709–714. 28105338 10.3892/br.2016.782 PMC5228363

[131] Mohammadhosayni M, , Matrix metalloproteinases are involved in the development of neurological complications in patients with Coronavirus disease 2019, Int Immunopharmacol. 100 (2021) 108076. 34450402 10.1016/j.intimp.2021.108076 PMC8367754

[132] Heymans S, , Inhibition of plasminogen activators or matrix metalloproteinases prevents cardiac rupture but impairs therapeutic angiogenesis and causes cardiac failure, Nat. Med 5 (10) (1999) 1135–1142. 10502816 10.1038/13459

[133] Kim EM, Hwang O, Role of matrix metalloproteinase-3 in neurodegeneration, J. Neurochem 116 (1) (2011) 22–32. 21044079 10.1111/j.1471-4159.2010.07082.x

[134] Ghorbani S, Yong VW, The extracellular matrix as modifier of neuroinflammation and remyelination in multiple sclerosis, Brain 144 (7) (2021) 1958–1973. 33889940 10.1093/brain/awab059 PMC8370400

[135] Chunder R, , Identification of a novel role for matrix metalloproteinase-3 in the modulation of B cell responses in multiple sclerosis, Front Immunol. 13 (2022) 1025377. 36389698 10.3389/fimmu.2022.1025377 PMC9644161

[136] Aksnes M, , Sex-specific associations of matrix metalloproteinases in Alzheimer’s disease, Biol. Sex. Differ 14 (1) (2023) 35. 37221606 10.1186/s13293-023-00514-x PMC10207710

[137] Pentz R, , Nerve growth factor (NGF) pathway biomarkers in Down syndrome prior to and after the onset of clinical Alzheimer’s disease: a paired CSF and plasma study, Alzheimers Dement 17 (4) (2021) 605–617. 33226181 10.1002/alz.12229 PMC8043977

[138] Mroczko B, , Concentrations of matrix metalloproteinases and their tissue inhibitors in the cerebrospinal fluid of patients with Alzheimer’s disease, J. Alzheimers Dis 40 (2) (2014) 351–357. 24448781 10.3233/JAD-131634

[139] Hanzel CE, , Analysis of matrix metallo-proteases and the plasminogen system in mild cognitive impairment and Alzheimer’s disease cerebrospinal fluid, J. Alzheimers Dis 40 (3) (2014) 667–678. 24531161 10.3233/JAD-132282

[140] Stomrud E, , Alterations of matrix metalloproteinases in the healthy elderly with increased risk of prodromal Alzheimer’s disease, Alzheimers Res Ther. 2 (3) (2010) 20. 20576109 10.1186/alzrt44 PMC2919700

[141] Horstmann S, , Matrix metalloproteinases in peripheral blood and cerebrospinal fluid in patients with Alzheimer’s disease, Int Psychogeriatr. 22 (6) (2010) 966–972. 20561382 10.1017/S1041610210000827

[142] Zhang WJ, , Clinical features and potential mechanisms relating neuropathological biomarkers and blood-brain barrier in patients with Alzheimer’s disease and hearing loss, Front Aging Neurosci. 14 (2022) 911028. 35783139 10.3389/fnagi.2022.911028 PMC9245454

[143] Mlekusch R, Humpel C, Matrix metalloproteinases-2 and −3 are reduced in cerebrospinal fluid with low beta-amyloid1-42 levels, Neurosci. Lett 466 (3) (2009) 135–138. 19786072 10.1016/j.neulet.2009.09.043 PMC4311377

[144] Yoshiyama Y, Asahina M, Hattori T, Selective distribution of matrix metalloproteinase-3 (MMP-3) in Alzheimer’s disease brain, Acta Neuropathol. 99 (2) (2000) 91–95. 10672313 10.1007/pl00007428

[145] Baig S, Kehoe PG, Love S, MMP-2, −3 and −9 levels and activity are not related to Abeta load in the frontal cortex in Alzheimer’s disease, Neuropathol. Appl. Neurobiol 34 (2) (2008) 205–215. 17971072 10.1111/j.1365-2990.2007.00897.x

[146] Spindola A, , Increased Mmp/reck expression ratio is associated with increased recognition memory performance in a Parkinson’s disease animal model, Mol. Neurobiol 57 (2) (2020) 837–847. 31493243 10.1007/s12035-019-01740-4

[147] Liu CZ, , Correlation of matrix metalloproteinase 3 and matrix metalloproteinase 9 levels with non-motor symptoms in patients with Parkinson’s disease, Front Aging Neurosci. 14 (2022) 889257. 36072482 10.3389/fnagi.2022.889257 PMC9444063

[148] Shin EJ, , Matrix metalloproteinase-3 is activated by HtrA2/Omi in dopaminergic cells: relevance to Parkinson’s disease, Neurochem. Int 60 (3) (2012) 249–256. 22265821 10.1016/j.neuint.2012.01.001

[149] Kortekaas R, , Blood-brain barrier dysfunction in parkinsonian midbrain in vivo, Ann. Neurol 57 (2) (2005) 176–179. 15668963 10.1002/ana.20369

[150] Choi DH, , Role of matrix metalloproteinase 3-mediated alpha-synuclein cleavage in dopaminergic cell death, J. Biol. Chem 286 (16) (2011) 14168–14177. 21330369 10.1074/jbc.M111.222430 PMC3077618

[151] Aerts J, , Altered neuronal architecture and plasticity in the visual cortex of adult MMP-3-deficient mice, Brain Struct. Funct 220 (5) (2015) 2675–2689. 24957860 10.1007/s00429-014-0819-4

[152] Suhaimi SA, Chan SC, Rosli R, Matrix metallopeptidase 3 polymorphisms: emerging genetic markers in human breast cancer metastasis, J. Breast Cancer 23 (1) (2020) 1–9. 32140265 10.4048/jbc.2020.23.e17 PMC7043940

[153] Duffy MJ, , Metalloproteinases: role in breast carcinogenesis, invasion and metastasis, Breast Cancer Res 2 (4) (2000) 252–257. 11250717 10.1186/bcr65 PMC138784

[154] Ongusaha PP, , HB-EGF is a potent inducer of tumor growth and angiogenesis, Cancer Res 64 (15) (2004) 5283–5290. 15289334 10.1158/0008-5472.CAN-04-0925

[155] Lynch CC, Matrisian LM, Matrix metalloproteinases in tumor-host cell communication, Differentiation 70 (9–10) (2002) 561–573. 12492497 10.1046/j.1432-0436.2002.700909.x

[156] Taha EA, , Knockout of MMP3 weakens solid tumor organoids and cancer extracellular vesicles, Cancers 12 (5) (2020). 10.3390/cancers12051260 PMC7281240 32429403

[157] Krippl P, , The 5A/6A polymorphism of the matrix metalloproteinase 3 gene promoter and breast cancer, Clin. Cancer Res 10 (10) (2004) 3518–3520. 15161710 10.1158/1078-0432.CCR-04-0010

[158] Deb G, , Epigenetic induction of tissue inhibitor of matrix metalloproteinase-3 by green tea polyphenols in breast cancer cells, Mol. Carcinog 54 (6) (2015) 485–499. 24481780 10.1002/mc.22121

[159] Sternlicht MD, , The stromal proteinase MMP3/stromelysin-1 promotes mammary carcinogenesis, Cell 98 (2) (1999) 137–146. 10428026 10.1016/s0092-8674(00)81009-0 PMC2853255

[160] Argote Camacho AX, , Metalloproteinases 1 and 3 as potential biomarkers in breast cancer development, Int J. Mol. Sci 22 (16) (2021). 10.3390/ijms22169012 PMC8396449 34445715

[161] Ławicki P, , Plasma levels of metalloproteinase 3 (MMP-3) and metalloproteinase 7 (MMP-7) as new candidates for tumor biomarkers in diagnostic of breast cancer patients, J. Clin. Med 12 (7) (2023). 10.3390/jcm12072618 PMC10094779 37048701

[162] Cai M, , Overexpression of angiogenic factors and matrix metalloproteinases in the saliva of oral squamous cell carcinoma patients: potential non-invasive diagnostic and therapeutic biomarkers, BMC Cancer 22 (1) (2022) 530. 35545767 10.1186/s12885-022-09630-0 PMC9092712

[163] Polz A, , Could MMP3 and MMP9 serve as biomarkers in EBV-related oropharyngeal cancer, Int J. Mol. Sci 25 (5) (2024). 10.3390/ijms25052561 PMC10931672 38473807

[164] Frieling JS, , Prostate cancer-derived MMP-3 controls intrinsic cell growth and extrinsic angiogenesis, Neoplasia 22 (10) (2020) 511–521. 32896761 10.1016/j.neo.2020.08.004 PMC7481881

[165] Kader AK, , Matrix metalloproteinase polymorphisms and bladder cancer risk, Cancer Res 66 (24) (2006) 11644–11648. 17178858 10.1158/0008-5472.CAN-06-1212

[166] Srivastava P, , Role of MMP-3 and MMP-9 and their haplotypes in risk of bladder cancer in North Indian cohort, Ann. Surg. Oncol 17 (11) (2010) 3068–3075. 20574775 10.1245/s10434-010-1153-6

[167] El-Sharkawi F, , The biochemical value of urinary metalloproteinases 3 and 9 in diagnosis and prognosis of bladder cancer in Egypt, J. Biomed. Sci 21 (1) (2014) 72. 25135219 10.1186/s12929-014-0072-4 PMC4237805

[168] Ay A, Alkanli N, Cevik G, Investigation of the relationship between MMP-1 (−1607 1G/2G), MMP-3 (- 1171 5A/6A) gene variations and development of bladder cancer, Mol. Biol. Rep 48 (12) (2021) 7689–7695. 34693500 10.1007/s11033-021-06775-2

[169] Chen W, , Over-expression of USP15/MMP3 predict poor prognosis and promote growth, migration in non-small cell lung cancer cells, Cancer Genet 272–273 (2023) 9–15. 10.1016/j.cancergen.2023.01.001 36640492

[170] Cymbaluk-Płoska A, , Suitability assessment of baseline concentration of MMP3, TIMP3, HE4 and CA125 in the serum of patients with ovarian cancer, J. Ovarian Res 11 (1) (2018) 1. 29304854 10.1186/s13048-017-0373-9 PMC5755423

[171] Chuliá-Peris L, , Matrix metalloproteinases and their inhibitors in pulmonary fibrosis: EMMPRIN/CD147 comes into play, Int J. Mol. Sci 23 (13) (2022). 10.3390/ijms23136894 PMC9267107 35805895

[172] Puntorieri V, , Lack of matrix metalloproteinase 3 in mouse models of lung injury ameliorates the pulmonary inflammatory response in female but not in male mice, Exp. Lung Res 42 (7) (2016) 365–379. 27676418 10.1080/01902148.2016.1231243

[173] Bauer A, Habior A, Concentration of serum matrix metalloproteinase-3 in patients with primary biliary cholangitis, Front Immunol. 13 (2022) 885229. 35529854 10.3389/fimmu.2022.885229 PMC9072739

[174] Fu Y, , Clinical value of serum MMP-3 in chronic kidney disease, Clin. Chim. Acta 553 (2024) 117725. 38128817 10.1016/j.cca.2023.117725

[175] Inatomi O, , Matrix metalloproteinase-3 secretion from human pancreatic periacinar myofibroblasts in response to inflammatory mediators, Pancreas 34 (1) (2007) 126–132. 17198194 10.1097/01.mpa.0000246662.23128.57

[176] Page-McCaw A, Ewald AJ, Werb Z, Matrix metalloproteinases and the regulation of tissue remodelling, Nat. Rev. Mol. Cell Biol 8 (3) (2007) 221–233. 17318226 10.1038/nrm2125 PMC2760082

[177] Suzuki M, , Matrix metalloproteinase-3 releases active heparin-binding EGF-like growth factor by cleavage at a specific juxtamembrane site, J. Biol. Chem 272 (50) (1997) 31730–31737. 9395517 10.1074/jbc.272.50.31730

[178] Domeij H, Yucel-Lindberg T, Modéer T, Signal pathways involved in the production of MMP-1 and MMP-3 in human gingival fibroblasts, Eur. J. Oral. Sci 110 (4) (2002) 302–306. 12206592 10.1034/j.1600-0722.2002.21247.x

[179] Maeda S, , The first stage of transforming growth factor beta1 activation is release of the large latent complex from the extracellular matrix of growth plate chondrocytes by matrix vesicle stromelysin-1 (MMP-3), Calcif. Tissue Int 70 (1) (2002) 54–65. 11907708 10.1007/s002230010032

[180] Maeda S, , Activation of latent transforming growth factor beta1 by stromelysin 1 in extracts of growth plate chondrocyte-derived matrix vesicles, J. Bone Min. Res 16 (7) (2001) 1281–1290. 10.1359/jbmr.2001.16.7.1281 11450704

[181] Liao J, , Cross-talk between the TGF-β and cell adhesion signaling pathways in cancer, Int J. Med Sci 21 (7) (2024) 1307–1320. 38818471 10.7150/ijms.96274 PMC11134594

[182] Meng XM, Nikolic-Paterson DJ, Lan HY, TGF-β: the master regulator of fibrosis, Nat. Rev. Nephrol 12 (6) (2016) 325–338. 27108839 10.1038/nrneph.2016.48

[183] Derynck R, Budi EH, Specificity, versatility, and control of TGF-β family signaling, Sci. Signal 12 (570) (2019). 10.1126/scisignal.aav5183 PMC6800142 30808818

[184] Anastas JN, Moon RT, WNT signalling pathways as therapeutic targets in cancer, Nat. Rev. Cancer 13 (1) (2013) 11–26. 23258168 10.1038/nrc3419

[185] Nusse R, Clevers H, Wnt/β-catenin signaling, disease, and emerging therapeutic modalities, Cell 169 (6) (2017) 985–999. 28575679 10.1016/j.cell.2017.05.016

[186] Kessenbrock K, , A role for matrix metalloproteinases in regulating mammary stem cell function via the Wnt signaling pathway, Cell Stem Cell 13 (3) (2013) 300–313. 23871604 10.1016/j.stem.2013.06.005 PMC3769456

[187] Russell SB, , Epigenetically altered wound healing in keloid fibroblasts, J. Invest Dermatol 130 (10) (2010) 2489–2496. 20555348 10.1038/jid.2010.162 PMC2939920

[188] Hirata H, , Role of secreted frizzled-related protein 3 in human renal cell carcinoma, Cancer Res 70 (5) (2010) 1896–1905. 20160027 10.1158/0008-5472.CAN-09-3549

[189] Blavier L, , Stromelysin-1 (MMP-3) is a target and a regulator of Wnt1-induced epithelial-mesenchymal transition (EMT), Cancer Biol. Ther 10 (2) (2010) 198–208. 20534975 10.4161/cbt.10.2.12193 PMC3040898

[190] Bougault C, , Protective role of frizzled-related protein B on matrix metalloproteinase induction in mouse chondrocytes, Arthritis Res Ther 16 (4) (2014) R137. 24984954 10.1186/ar4599 PMC4226985

[191] Nieto MA, , EMT: 2016, Cell 166 (1) (2016) 21–45. 27368099 10.1016/j.cell.2016.06.028

[192] Dongre A, Weinberg RA, New insights into the mechanisms of epithelial-mesenchymal transition and implications for cancer, Nat. Rev. Mol. Cell Biol 20 (2) (2019) 69–84. 30459476 10.1038/s41580-018-0080-4

[193] Jinesh GG, Brohl AS, Classical epithelial-mesenchymal transition (EMT) and alternative cell death process-driven blebbishield metastatic-witch (BMW) pathways to cancer metastasis, Signal Transduct. Target Ther 7 (1) (2022) 296. 35999218 10.1038/s41392-022-01132-6 PMC9399134

[194] Yu Y, , Cancer-associated fibroblasts induce epithelial-mesenchymal transition of breast cancer cells through paracrine TGF-β signalling, Br. J. Cancer 110 (3) (2014) 724–732. 24335925 10.1038/bjc.2013.768 PMC3915130

[195] Stemmer V, , Snail promotes Wnt target gene expression and interacts with beta-catenin, Oncogene 27 (37) (2008) 5075–5080. 18469861 10.1038/onc.2008.140

[196] Yuan X, , Notch signaling and EMT in non-small cell lung cancer: biological significance and therapeutic application, J. Hematol. Oncol 7 (2014) 87. 25477004 10.1186/s13045-014-0087-z PMC4267749

[197] Lochter A, , Matrix metalloproteinase stromelysin-1 triggers a cascade of molecular alterations that leads to stable epithelial-to-mesenchymal conversion and a premalignant phenotype in mammary epithelial cells, J. Cell Biol 139 (7) (1997) 1861–1872. 9412478 10.1083/jcb.139.7.1861 PMC2132651

[198] Lochter A, , Misregulation of stromelysin-1 expression in mouse mammary tumor cells accompanies acquisition of stromelysin-1-dependent invasive properties, J. Biol. Chem 272 (8) (1997) 5007–5015. 9030563 10.1074/jbc.272.8.5007

[199] Pelisch F, , Involvement of hnRNP A1 in the matrix metalloprotease-3-dependent regulation of Rac1 pre-mRNA splicing, J. Cell Biochem 113 (7) (2012) 2319–2329. 22345078 10.1002/jcb.24103 PMC3927408

[200] Radisky DC, , Rac1b and reactive oxygen species mediate MMP-3-induced EMT and genomic instability, Nature 436 (7047) (2005) 123–127. 16001073 10.1038/nature03688 PMC2784913

[201] Fowlkes JL, , Regulation of insulin-like growth factor (IGF)-I action by matrix metalloproteinase-3 involves selective disruption of IGF-I/IGF-binding protein-3 complexes, Endocrinology 145 (2) (2004) 620–626. 14605000 10.1210/en.2003-0636

[202] Blumenfeld I, , Metalloproteinases (MMPs −2, −3) are involved in TGF-beta and IGF-1-induced bone defect healing in 20-month-old female rats, Arch. Gerontol. Geriatr 35 (1) (2002) 59–69. 14764345 10.1016/s0167-4943(02)00004-3

[203] Sani M, , Engineered artificial articular cartilage made of decellularized extracellular matrix by mechanical and IGF-1 stimulation, Biomater. Adv 139 (2022) 213019. 35882114 10.1016/j.bioadv.2022.213019

[204] Wouters MA, , Evolution of distinct EGF domains with specific functions, Protein Sci. 14 (4) (2005) 1091–1103. 15772310 10.1110/ps.041207005 PMC2253431

[205] Gogou C, , Alternative splicing controls teneurin-3 compact dimer formation for neuronal recognition, Nat. Commun 15 (1) (2024) 3648. 38684645 10.1038/s41467-024-47763-x PMC11058771

[206] Hynes NE, Lane HA, ERBB receptors and cancer: the complexity of targeted inhibitors, Nat. Rev. Cancer 5 (5) (2005) 341–354. 15864276 10.1038/nrc1609

[207] Shu J, , Matrix metalloproteinase 3 regulates angiotensin II-induced myocardial fibrosis cell viability, migration and apoptosis, Mol. Med Rep 23 (2) (2021). 10.3892/mmr.2020.11790 PMC7789094 33655326

[208] Manka SW, Bihan D, Farndale RW, Structural studies of the MMP-3 interaction with triple-helical collagen introduce new roles for the enzyme in tissue remodelling, Sci. Rep 9 (1) (2019) 18785. 31827179 10.1038/s41598-019-55266-9 PMC6906530

[209] Ågren MS, , Tumor necrosis factor-α-accelerated degradation of type I collagen in human skin is associated with elevated matrix metalloproteinase (MMP)-1 and MMP-3 ex vivo, Eur. J. Cell Biol 94 (1) (2015) 12–21. 25457675 10.1016/j.ejcb.2014.10.001 PMC4300401

[210] Jobin PG, Butler GS, Overall CM, New intracellular activities of matrix metalloproteinases shine in the moonlight, Biochim Biophys. Acta Mol. Cell Res 1864 (11 Pt A) (2017) 2043–2055. 28526562 10.1016/j.bbamcr.2017.05.013

[211] Eguchi T, , Novel transcription-factor-like function of human matrix metalloproteinase 3 regulating the CTGF/CCN2 gene, Mol. Cell Biol 28 (7) (2008) 2391–2413. 18172013 10.1128/MCB.01288-07 PMC2268440

[212] Si-Tayeb K, , Matrix metalloproteinase 3 is present in the cell nucleus and is involved in apoptosis, Am. J. Pathol 169 (4) (2006) 1390–1401. 17003494 10.2353/ajpath.2006.060005 PMC1780186

[213] Yu WH, , CD44 anchors the assembly of matrilysin/MMP-7 with heparin-binding epidermal growth factor precursor and ErbB4 and regulates female reproductive organ remodeling, Genes Dev. 16 (3) (2002) 307–323. 11825873 10.1101/gad.925702 PMC155329

[214] Emonard H, , Low density lipoprotein receptor-related protein mediates endocytic clearance of pro-MMP-2.TIMP-2 complex through a thrombospondin-independent mechanism, J. Biol. Chem 279 (52) (2004) 54944–54951. 15489233 10.1074/jbc.M406792200

[215] Zuo X, , Matrix metalloproteinase 3 promotes cellular anti-dengue virus response via interaction with transcription factor NFκB in cell nucleus, PLoS One 9 (1) (2014) e84748. 24416274 10.1371/journal.pone.0084748 PMC3885614

[216] Massagué J, TGFβ signalling in context, Nat. Rev. Mol. Cell Biol 13 (10) (2012) 616–630. 22992590 10.1038/nrm3434 PMC4027049

[217] David CJ, Massagúe J, Contextual determinants of TGFβ action in development, immunity and cancer, Nat. Rev. Mol. Cell Biol 19 (7) (2018) 419–435. 29643418 10.1038/s41580-018-0007-0 PMC7457231

